# Text messaging and brief phone calls for weight loss in overweight and obese English- and Spanish-speaking adults: A 1-year, parallel-group, randomized controlled trial

**DOI:** 10.1371/journal.pmed.1002917

**Published:** 2019-09-25

**Authors:** Job G. Godino, Natalie M. Golaszewski, Greg J. Norman, Cheryl L. Rock, William G. Griswold, Elva Arredondo, Simon Marshall, Julie Kolodziejczyk, Lindsay Dillon, Fred Raab, Sonia Jain, Maggie Crawford, Gina Merchant, Kevin Patrick

**Affiliations:** 1 Center for Wireless and Population Health Systems, University of California, San Diego, La Jolla, California, United States of America; 2 Department of Family Medicine and Public Health, University of California, San Diego, La Jolla, California, United States of America; 3 Exercise and Physical Activity Resource Center, University of California, San Diego, La Jolla, California, United States of America; 4 Department of Computer Science and Engineering, University of California, San Diego, La Jolla, California, United States of America; 5 Graduate School of Public Health, San Diego State University, San Diego, California, United States of America; Harvard Medical School, UNITED STATES

## Abstract

**Background:**

Weight loss interventions based solely on text messaging (short message service [SMS]) have been shown to be modestly effective for short periods of time and in some populations, but limited evidence is available for positive longer-term outcomes and for efficacy in Hispanic populations. Also, little is known about the comparative efficacy of weight loss interventions that use SMS coupled with brief, technology-mediated contact with health coaches, an important issue when considering the scalability and cost of interventions. We examined the efficacy of a 1-year intervention designed to reduce weight among overweight and obese English- and Spanish-speaking adults via SMS alone (ConTxt) or in combination with brief, monthly health-coaching calls. ConTxt offered 2–4 SMS/day that were personalized, tailored, and interactive. Content was theory- and evidence-based and focused on reducing energy intake and increasing energy expenditure. Monthly health-coaching calls (5–10 minutes’ duration) focused on goal-setting, identifying barriers to achieving goals, and self-monitoring.

**Methods and findings:**

English- and Spanish-speaking adults were recruited from October 2011 to March 2013. A total of 298 overweight (body mass index [BMI] 27.0 to 39.9 kg/m^2^) adults (aged 21–60 years; 77% female; 41% Hispanic; 21% primarily Spanish speaking; 44% college graduates or higher; 22% unemployed) were randomly assigned (1:1) to receive either ConTxt only (*n* = 101), ConTxt plus health-coaching calls (*n* = 96), or standard print materials on weight reduction (control group, *n* = 101). We used computer-based permuted-block randomization with block sizes of three or six, stratified by sex and Spanish-speaking status. Participants, study staff, and investigators were masked until the intervention was assigned. The primary outcome was objectively measured percent of weight loss from baseline at 12 months. Differences between groups were evaluated using linear mixed-effects regression within an intention-to-treat framework. A total of 261 (87.2%) and 253 (84.9%) participants completed 6- and 12-month visits, respectively. Loss to follow-up did not differ by study group. Mean (95% confidence intervals [CIs]) percent weight loss at 12 months was −0.61 (−1.99 to 0.77) in the control group, −1.68 (−3.08 to −0.27) in ConTxt only, and −3.63 (−5.05 to −2.81) in ConTxt plus health-coaching calls. At 12 months, mean (95% CI) percent weight loss, adjusted for baseline BMI, was significantly different between ConTxt plus health-coaching calls and the control group (−3.0 [−4.99 to −1.04], *p* = 0.003) but not between the ConTxt-only and the control group (−1.07 [−3.05 to 0.92], *p* = 0.291). Differences between ConTxt plus health-coaching calls and ConTxt only were not significant (−1.95 [−3.96 to 0.06], *p* = 0.057). These findings were consistent across other weight-related secondary outcomes, including changes in absolute weight, BMI, and percent body fat at 12 months. Exploratory subgroup analyses suggested that Spanish speakers responded more favorably to ConTxt plus health-coaching calls than English speakers (Spanish contrast: −7.90 [−11.94 to −3.86], *p* < 0.001; English contrast: −1.82 [−4.03 to 0.39], *p* = 0.107). Limitations include the unblinded delivery of the intervention and recruitment of a predominantly female sample from a single site.

**Conclusions:**

A 1-year intervention that delivered theory- and evidence-based weight loss content via daily personalized, tailored, and interactive SMS was most effective when combined with brief, monthly phone calls.

**Trial registration:**

ClinicalTrials.gov NCT01171586

## Author summary

**Why was this study done?:**

- Overweight and obese adults have increased risk for several chronic diseases, including type 2 diabetes, hypertension, cardiovascular disease, and many types of cancer.
- Most effective weight loss interventions are costly, require in-person behavioral counseling, and lack evidence of long-term effectiveness.
- We sought to determine whether weight loss content delivered by text message alone or in combination with brief, technology-mediated contact with health coaches that is scalable can improve weight loss among English- and Spanish-speaking adults.

**What did the researchers do and find?:**

- We examined the efficacy of a 1-year randomized controlled trial designed to reduce weight among overweight and obese English- and Spanish-speaking adults via theory- and evidence-based, personalized, tailored, and interactive text messaging alone (ConTxt) or in combination with brief, monthly health-coaching calls.
- At 12 months, mean percent weight loss, adjusted for baseline body mass index, was significantly different between ConTxt plus health-coaching calls and the control group but not between ConTxt only and the control group. The combined intervention resulted in a weight loss equal to 3.6%, contrasted against those in the control who lost 0.6%, at 1 year among a diverse sample of English- and Spanish-speaking adults who were overweight or obese.

**What do these findings mean?:**

- A 1-year intervention that delivered theory- and evidence-based weight loss content via daily personalized, tailored, and interactive text messaging was most effective when combined with brief, monthly phone calls.
- Given the high scalability of our intervention, these results represent an intriguing weight loss approach, particularly among hard-to-reach populations who are disproportionately affected by the negative consequences of overweight and obesity.

## Introduction

Overweight and obese adults have increased risk for several chronic diseases, including type 2 diabetes, hypertension, cardiovascular disease, and many types of cancer [1]. In 2014, costs associated with the growing obesity epidemic in the United States totaled $149.4 billion [2]. Hispanics are at increased risk for overweight and obesity compared to non-Hispanics and suffer disproportionately from obesity-related comorbidities [3,4]. Fortunately, research has shown that among those who are overweight and obese, a weight loss of 5% of body weight substantially decreases chronic disease risk [1]. However, most effective weight loss interventions are costly, require in-person behavioral counseling, and lack evidence of long-term effectiveness [5,6]. Furthermore, few have been evaluated in Hispanic populations, which account for 42.5% of the obese population and comprise the largest ethnic minority group in the USA [3,4,7].

Interventions using text messaging (i.e., short message service [SMS]) have begun to show increasing promise for the management of overweight and obesity. SMS is convenient and accessible, as there is near-universal mobile phone ownership within the USA [8], including within communities of low socioeconomic status [9]. SMS interventions enable the ability to prompt and reward healthy behaviors in near real-time and allow two-way interactions between intervention participants and health coaches [10]. SMS interventions that combine personalization with extended, frequent interactions have been shown to support long-term adherence to self-monitoring, a behavior-change technique shown to be effective in promoting weight loss [6,11,12].

To date, weight loss interventions utilizing SMS have typically included small sample sizes [13–15] and only 1 to 6 months of intervention contact [13,14,16,17]. Several previous SMS weight loss interventions have also incorporated extensive contact with health coaches [14,18,19], which is costly in regard to time and personnel. It remains unclear whether weight loss content delivered by SMS alone is effective or whether similar intervention content coupled with brief, technology-mediated contact with health coaches that is scalable can optimize weight loss. Additionally, few studies have included samples of racial or ethnic minorities. Given that Hispanics have similar rates as non-Hispanics (96%) of owning mobile phones [20], and they are more likely to use their phone for information about a health condition [21], SMS represents a promising intervention modality among this population.

In the present study, we examined the efficacy of a 1-year intervention designed to reduce weight among overweight and obese English- and Spanish-speaking adults through theory- and evidence-based weight loss content delivered via SMS (ConTxt) only or in combination with brief, monthly health-coaching calls. We hypothesized that compared to providing standard weight loss information via printed materials (a control condition), ConTxt would lead to clinically meaningful weight loss after 1 year. Also, we hypothesized that the inclusion of brief, monthly health-coaching calls would result in more weight loss than ConTxt only would. Secondary behavioral and psychological outcomes were also measured in this study. They include diet, sedentary behavior, physical activity, sleep, self-esteem, body image, and well-being. The intervention effects on these outcomes will be reported elsewhere.

## Methods

### Study design

We conducted a 1-year, parallel-group, randomized controlled trial among overweight and obese adults (body mass index [BMI] 27.0 to 39.9 kg/m^2^) in San Diego, CA, USA. The study procedures were approved by the University of California, San Diego Institutional Review Board. Each participant provided written informed consent. The trial is registered with ClinicalTrials.gov (NCT01171586; date posted: July 28, 2010). The study protocol and CONSORT checklist can be found in the supporting information.

### Participant recruitment and screening

English- and Spanish-speaking adults were recruited from October 2011 to March 2013 via print advertisements, email listservs, and online community boards. Potential participants were screened via telephone by bilingual recruitment staff for meeting the following inclusion criteria: 21 to 60 years old, overweight (BMI 27.0 to 39.9 kg/m^2^), owner of a cell phone capable of sending and receiving SMS, and residency in San Diego County. Exclusion criteria included experiencing pulmonary, cardiovascular, or musculoskeletal problems that would limit the ability to comply with study protocols; using medications that altered weight; having a history of eating disorders, weight loss surgery, substance abuse, or psychiatric disorders; being a smoker or recently quitting smoking; participating in another weight loss program; living with someone enrolled in the study; and being pregnant or intending to become pregnant. The Physical Activity Readiness Questionnaire (PAR-Q) was used to screen potential participants’ ability to engage in moderate physical activity [22]. Potential participants who answered “yes” to at least one of the PAR-Q questions were required to obtain approval to participate in the study from a licensed medical doctor.

### Randomization and masking

After completing a baseline measurement visit, a statistician (GN) allocated participants (1:1) to the intervention groups or control group using computer-based permuted-block randomization with block sizes of three or six that were stratified by sex and Spanish-speaking status. Allocation was concealed from the participants, study staff, and investigators until the intervention was assigned. It was not possible to mask participants or the study staff and health coaches responsible for delivering the intervention components. However, study staff who measured participants and investigators who analyzed study outcomes remained masked to the allocation throughout the study.

### Procedures

**Fig 1:**
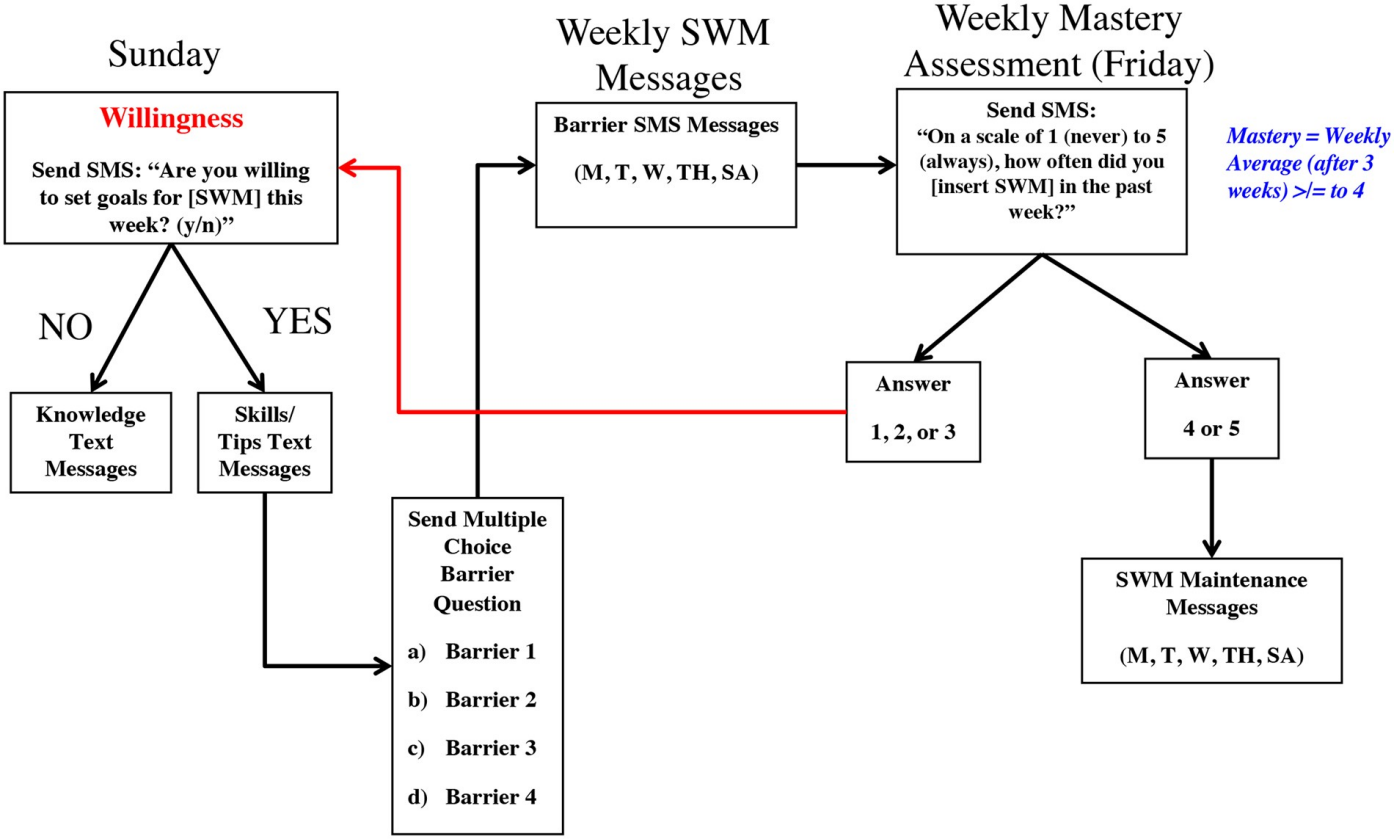
Text message (SMS) logic and flow of tailored messages. SMS, short message service.

The ConTxt intervention consisted of approximately 2,100 English and 2,100 Spanish SMS, many of which were personalized, tailored, and interactive throughout the length of the intervention (Table 1 contains detailed descriptions of the features of the message content). Daily engagement was measured through likes/dislikes or no response to the daily SMS. Messages were provided in either English or Spanish based on stated preferences. SMS contained theory- and evidence-based weight loss content (Table 2 lists example SMS and the theory or evidence basis for the content). A large portion of messages focused on content derived from the Strategies for Weight Management (SWM) Inventory [23,24], which is a 35-item questionnaire that assesses use of recommended behavioral strategies for reducing energy intake and increasing energy expenditure in overweight and obese adults. Programming logic was developed to maximize participant preferences for SMS content and recognize progress toward mastering the adoption of behaviors in the SWM (Fig 1 shows the logic and flow of tailored messages targeting behaviors in the SWM). The ConTxt intervention was designed to work with the Omron HJ-150 pedometer, which was provided to participants at no cost at the start of the intervention. Study staff explained how to use the pedometer, how to convert nonpedometric activities or minutes of exercise to steps, and how to respond to the SMS “how many steps did you achieve yesterday?”

**Table 1:** Description of the features of the ConTxt intervention. Abbreviations: SMS, short message service; SWM, Strategies for Weight Management.

Personalization, tailoring, or interaction	Description
Time of messages	Participants were able to select four times that messages were delivered on each day of the week. For examples, users could have their first message sent early on weekdays and later in the day on weekends. The SMS system processed this information to only send content during these four times.
Frequency of messages	Participants had the option at 4 months, 6 months, and 8 months to change the number of messages they received. For example, they received via an SMS message, “For the next 4 months, would you like to the number of messages you get to A) stay the same D) decrease a little bit or G) decrease a lot?” The SMS system then adjusted the frequency of messages based on the participants’ responses at each time point.
Like/unlike	Participants were able to influence the types of messages they received. They were encouraged to “like” or “not like” messages. The “like”and “not like” system allowed users to receive more content that they liked and less of the content that they did not like.
Feedback	The SMS system provided feedback (i.e., a response) to any message that a user sent to the SMS system. For example, if a user reported their weight, which was requested weekly, the user would receive feedback based on their weight loss/gain from the previous week. The SMS system also provided a weekly step count average once a week.
Milestones	Milestone messages were sent when a participant reached a weight loss milestone. For example, the SMS system would send a message when they lost 5 lbs or 20 lbs, 10% of body weight, or a specific cumulative step count milestone (i.e., “Your cumulative step count is equivalent to walking from San Diego to San Francisco”).
Competitive messages	The SMS system would send participants competitive messages that compared their average step count or weight loss to that of other study participants.
Personalized text embedded within a SMS message	Participants were asked a series of questions about friends or family who will support them during the weight loss journey, their favorite restaurants, name of a grocery store where they shop, and name of nearby park. These text fields were then embedded into relevant SMS content.
Physical activity and location preference	Participants were asked to select their preferred type of physical activity from a list of preselected activities: walk, run, bike, swim, exercise class, or other. They were also asked to select their preferred location for doing physical activity from a list of preselected locations: home, gym, outside, office. With this information, the SMS system would send SMS content related to their preferred activity and location type.
SWM	The SWM Inventory was administered at baseline (and 6 months) and used to identify unique weight management behavior challenges for each user. The SMS system processed these data to create goals to target based upon logic rules of the expert system.
Food temptation	Participants were asked to select a food temptation/craving from a list of preselected food cravings: chocolate, sweets, salty, comfort, general. The SMS system would send SMS content related to overcoming their cravings.
Participant-initiated messages	Participants could send the SMS system a preset keyword (name of a restaurant, breakfast, lunch, dinner, snack, chocolate, sweets, salty, comfort, home, work, gym, outside, run, walk, bike, swim) if they wanted content about one of the preselected keywords. For example, if a user was at Burger King and wanted an idea of a healthy option, the user could text “Burger King” and would receive a text message with a healthy option from the Burger King menu (including calories).

**Table 2:** Weekly topics and example messages.

Weekly topics	Example message	Theory or evidence basis
Self-monitoring and goal-setting	It’s important to track what you eat EVERY day. [FIRST NAME], remind yourself of your goals!	Behavioral self-monitoring and goal-setting prompts
Barriers	What is preventing you from following a structured meal plan? A) not organized D) I don’t know how G) no time J) I forget M) Other	Perceived barriers
Motivational messages	Do not let the small setbacks discourage you and instead use them as motivation to push yourself harder.	Social support and knowledge
Knowledge messages	Whenever you can, walk the long route or take the stairs rather than elevators to stay physically active.	Knowledge
Mastery	On a scale of 1 (never) to 5 (always), how often did you remove high calorie foods from your home, office, or room this week?	Behavioral skills
User-initiated database	Buying snacks from [GROCERY STORE] will usually be cheaper than stopping for fast food or the vending machine.	Tailoring and personalized feedback
Social situations	Don’t be alone in your endeavor to eat healthier. Encourage others to do so too! Why not hold a “healthy potluck?”	Social support
Calories	Pause and think about what you can do today to reduce your calories. Set your mind to it and do it.	Goal-setting prompts
Food cravings	Moderation! Try having just 5 dark chocolate chips. These have less fat and more antioxidants than milk chocolate.	Goal-setting prompts and knowledge
Step count	How many steps did you get yesterday? [Enter Number]	Behavioral self-monitoring
Portion size	On a scale of 1 (never) to 5 (always), how often did you reduce your portions this week?	Behavioral self-monitoring and mastery
Sedentary behaviors	Reduce your screen time. When at home, try replacing 30 mins of TV for a brisk walk. You will have more energy.	Behavioral skills
Physical activity routine	Set out your workout clothes or pack your workout bag the night before.	Behavioral skills
Eating out	When eating out, remember to request dressings on the side and only use them sparingly.	Behavioral skills and knowledge
Weight loss	You are taking the right steps to lose those pounds! What can you add in your daily regimen to help you lose weight?	Behavioral self-monitoring
Weight maintenance	You may see weight fluctuations from day to day, but stay focused on the overall trend on your weekly weight graphs.	Knowledge

Text messages were based on the SWM^23,24^. SWMs include evidence and social cognitive theory constructs.

Abbreviation: SWM, Strategies for Weight Management

In addition to the ConTxt intervention, one group of participants received two brief counseling calls from a health coach in the first month, followed by one call in subsequent months. Counseling calls were conducted in English or Spanish by trained health coaches with experience in nutrition and physical activity behavioral counseling. Calls lasted approximately 5 to 10 minutes and focused on goal-setting, identifying barriers to achieving goals, and self-monitoring.

Participants randomized to the control group received standard print materials related to weight loss that were comparable to what one would receive from community centers or nonprofit governmental organizations. Materials were provided in English or Spanish and included information on healthy diet, sedentary behavior, and physical activity. Participants also had the option to receive additional monthly tip sheets either through email or standard mail.

All participants were asked to attend in-person measurement visits at baseline, month 6 (M6), and month 12 (M12). Demographic information on age, sex, ethnicity (Hispanic, non-Hispanic), marital status, income, and Spanish-speaking status were self-reported through a survey collected at baseline. Trained study staff took anthropometric measurements. Weight was measured objectively (to the nearest 0.1 kg) using a calibrated digital scale (Seca 703, Seca, Hamburg, Germany). Height was measured objectively (to the nearest 0.1 cm) using a stadiometer (Seca 703). Both weight and height were measured with participants wearing lightweight clothes but without shoes, and two separate measurements were averaged. BMI was calculated as weight in kilograms divided by height in meters squared. Body fat percentage was assessed at baseline and M12 by dual-energy x-ray absorptiometry (DXA). DXA provides a whole-body scan measure of body composition as well as regional measurements of fat mass, lean mass, and bone mass.

Participants received an incentive of $75 at baseline, $50 at M6, and $75 at M12. They were also given $15 to cover the cost of transportation to each measurement visit. Participants randomized to receive ConTxt in both arms were also given $10 a month to cover the cost of receiving SMS. Participants randomized to the control group were given the opportunity to receive ConTxt at no cost for 4 months after completing the study; however, they were not compensated, nor did they receive the health coaching.

### Outcomes

The primary outcome was objectively measured percent weight loss from baseline weight at M12 measured in kilograms. Secondary outcomes reported in the present manuscript include absolute weight change in kilograms, BMI change, and percent body fat change at M12. Exploratory subgroup analyses evaluated the interaction effects of age, sex, ethnicity, and language on the primary outcome. Additional exploratory analyses evaluated the effect of the “dose” of the intervention on the primary outcome. More specifically, “dose” was defined as the mean percent of daily engagement (i.e., the number of messages responded to divided by the number of messages received, multiplied by 100).

### Statistical analyses

All statistical analyses were conducted using R version 3.1.1 (the R Foundation, Vienna, Austria) (http://www.r-project.org) and two-tailed *p*-values with the predefined cutoff for statistical significance set at 0.05.

An a priori power calculation was conducted to determine the sample size required to detect a difference in the primary outcome, percent weight loss at M12, between the ConTxt intervention groups and the control group using a *t* test with 80% statistical power (the family-wise alpha level for a two-tailed test was set at 0.025). The effect size d was determined using results from the mDiet pilot study [10]. The mDiet pilot study resulted in an estimated between-group difference of 3.2% weight loss at 12 months, a clinically meaningful amount [25], corresponding to a standardized effect size d of 0.49 [10]. Based on this and allowing for an attrition rate of 25%, we determined that approximately 309 participants were needed (or 103 participants per group).

Descriptive statistics (proportions, means, and SD) described key demographic characteristics. Differences between groups were assessed with linear mixed-effects regression models, adjusted for baseline BMI. Models were specified with a between-subject factor of treatment group, a within-subject factor of time treated categorically, and a treatment group × time interaction, as well as a random intercept by participant. Estimated marginal means and corresponding 95% confidence intervals (CIs) of outcomes were computed at each time point. The primary analysis was a test of between-group differences in percent weight loss from baseline weight at M12. All other analyses were considered secondary or exploratory. All analyses were done using an intention-to-treat framework and included all participants. Parameter estimates were based on maximum-likelihood estimation, which allows for the inclusion of participants with missing data. This approach increases power compared with a completers analysis, uses all available data, and is an appropriate method for handling missing data when the extent of missing data is relatively small and missing completely at random.

Preplanned exploratory subgroup analyses were conducted to assess whether age, sex, ethnicity, and language moderated the intervention effects on percent weight loss, by adding a multiplicative interaction term for each separately into the primary outcome model and by assessing between-group differences within each subgroup independently. An additional preplanned, exploratory analysis was done to test whether level of engagement affected percent weight loss in the intervention groups using mixed-effects regression adjusted for BMI and language.

## Results

**Fig 2:**
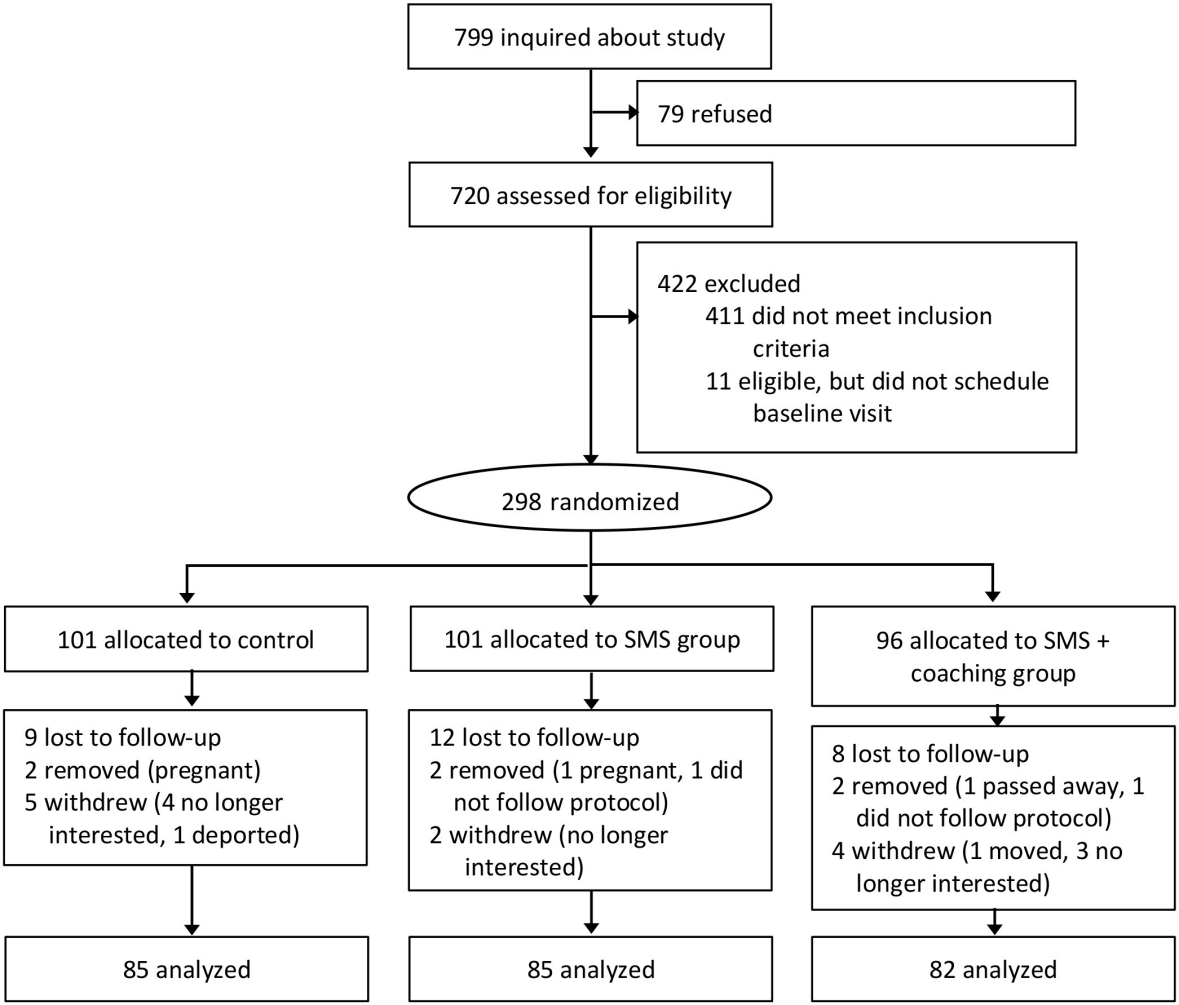
Flow of participants. SMS, short message service; SWM, Strategies for Weight Management.

The flow of participants through the study is shown in Fig 2. Of the 799 interested in the study, 298 were eligible and randomized to the following groups: ConTxt only (*n* = 101), ConTxt plus health-coaching calls (*n* = 96), and standard print materials (control group, *n* = 101). A total of 261 participants completed the M6 visit (87.2% completion rate), and 253 completed the M12 visit (84.9% completion rate). Seventeen participants withdrew or were removed from the study (e.g., became pregnant, no longer wanted to participate, did not comply with study protocols). Twenty-eight participants were lost to follow-up (e.g., unable to reach after numerous contact attempts via email, mail, phone, and text). Participants were 77% female, 41% Hispanic, 72% white, 21% primarily Spanish speaking, 44% college graduates or higher, 47% married, 18% in school, and 22% unemployed. Participants had a mean (SD) age of 41.7 (11.1) years and BMI of 32.7 (3.4) (Table 3).

**Table 3:** Baseline characteristics by study group.

Characteristics	Total (*n* = 298)	Standard print (*n* = 101)	SMS only (*n* = 101)	SMS + phone (*n* = 96)
Sex				
Female	228 (77%)	78 (77%)	77 (76%)	73 (76%)
Male	70 (24%)	23 (23%)	24 (24%)	23 (24%)
Language				
English	236 (79%)	79 (78%)	80 (79%)	77 (80%)
Spanish	62 (21%)	22 (22%)	21 (21%)	19 (20%)
Race				
White	155 (72%)	55 (75%)	51 (70%)	49 (71%)
Asian	8 (4%)	2 (3%)	4 (6%)	2 (3%)
Black	34 (16%)	10 (14%)	12 (16%)	12 (17%)
Other	18 (8%)	6 (8%)	6 (8%)	6 (9%)
Ethnicity				
Non-Hispanic	176 (59%)	59 (58%)	62 (61%)	55 (57%)
Hispanic	122 (41%)	42 (42%)	39 (39%)	41 (43%)
Education				
8th grade or less	12 (4%)	4 (4%)	4 (4%)	4 (4%)
Some high school	15 (5%)	2 (2%)	5 (5%)	8 (8%)
High school or GED	33 (11%)	11 (11%)	9 (9%)	13 (14%)
Trade or tech training	18 (6%)	9 (9%)	7 (7%)	2 (2%)
Some college	88 (30%)	26 (26%)	33 (33%)	29 (30%)
College graduate	65 (22%)	21 (21%)	26 (26%)	18 (19%)
Postgraduate training	18 (6%)	8 (8%)	7 (7%)	3 (3%)
Graduate degree	49 (16%)	20 (20%)	10 (10%)	19 (20%)
Marital Status				
Single	75 (25%)	24 (24%)	22 (22%)	29 (30%)
Married	139 (47%)	45 (45%)	49 (49%)	45 (47%)
Living with partner	23 (8%)	12 (12%)	7 (7%)	4 (4%)
Separated	7 (2%)	0 (0%)	2 (2%)	5 (5%)
Widowed	2 (1%)	1 (1%)	0 (0%)	1 (1%)
Divorced	52 (17%)	19 (19%)	21 (21%)	12 (13%)
Monthly Income				
Less than 1,000 USD	28 (9%)	8 (8%)	10 (10%)	10 (10%)
1,001–1,499 USD	39 (13%)	15 (15%)	9 (9%)	15 (16%)
1,500–1,999 USD	19 (6%)	5 (5%)	7 (7%)	7 (7%)
2,000–2,999 USD	36 (12%)	8 (8%)	11 (11%)	17 (18%)
3,000–3,999 USD	29 (10%)	14 (14%)	8 (8%)	7 (7%)
4,000–5,999 USD	43 (14%)	13 (13%)	20 (20%)	10 (10%)
6,000–8,500 USD	35 (12%)	10 (10%)	13 (13%)	12 (13%)
More than 8,500 USD	36 (12%)	15 (15%)	11 (11%)	10 (10%)
Preferred not to say	33 (11%)	13 (13%)	12 (12%)	8 (8%)
In School				
No	243 (82%)	81 (80%)	83 (82%)	79 (82%)
Yes	55 (19%)	20 (20%)	18 (18%)	17 (18%)
Employment				
No	66 (22%)	17 (17%)	27 (27%)	22 (23%)
Yes, full time	176 (59%)	63 (62%)	63 (62%)	50 (52%)
Yes, part time	56 (19%)	21 (21%)	11 (11%)	24 (25%)
Age	42 (11)	43 (11)	41 (11)	41 (11)
Weight (kg)	89 (12)	89 (14)	93 (14)	89 (12)
BMI (kg/m^2^)	33 (3)	32 (3)	34 (3)	32 (3)
Body composition (%fat)	46 (7)	46 (6)	45 (7)	45 (6)

*n* and percentages; mean and standard deviations for continuous variables.

Abbreviations: BMI, body mass index; SMS, short message service

**Fig 3:**
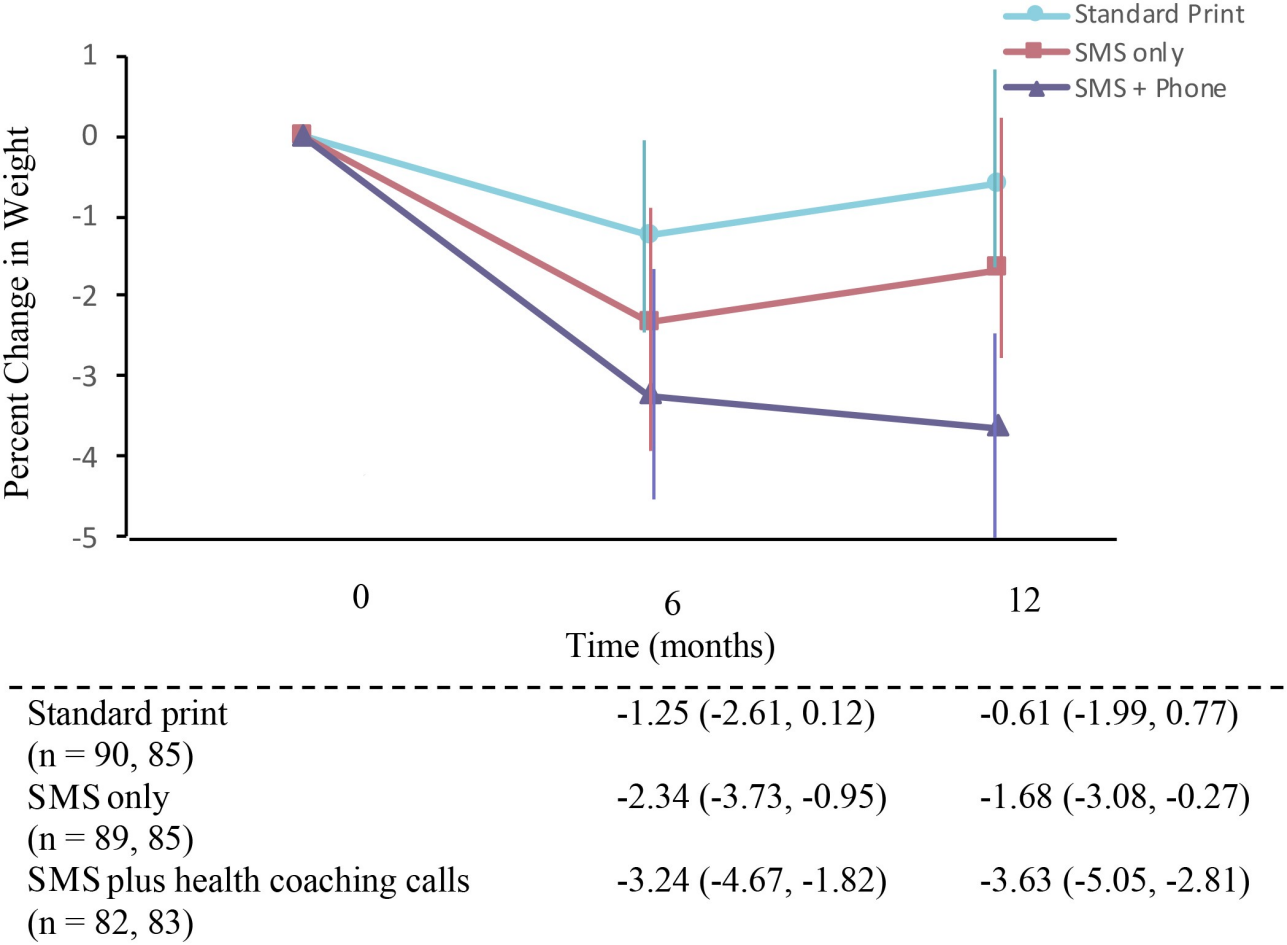
Estimated marginal means and 95% confidence intervals for the comparison of percent weight loss between ConTxt study groups over 12 months from linear mixed-effects regression model adjusted for baseline body mass index. SMS, short message service.

Fig 3 shows the estimated marginal means and 95% CIs for percent weight loss from baseline weight. There were no statistically significant differences in percent weight loss between the ConTxt-only and standard print groups at M6 (−1.10%, [95% CI −3.05 to 0.86], *p* = 0.27) or M12 (−1.07%, [95% CI −3.05 to 0.92], *p* = 0.29), nor were there differences between ConTxt only and ConTxt plus health-coaching call at M6 (−0.90%, [95% CI −2.91 to 1.10], *p* = 0.38) or M12 (−1.95%, [95% CI −3.96 to 0.06], *p* = 0.06). However, there were statistically significant differences in percent weight loss between the ConTxt plus health-coaching calls and standard print groups at M6 (−2.00%, [95% CI −3.96 to −0.03], *p* < 0.05) and M12 (−3.0%, [95% CI −4.99 to −1.04], *p* < 0.01). There was no evidence that effects were moderated by age, sex, and ethnicity (*p* > 0.05 for all interaction terms). Although the interaction term for language was not statistically significant (*p* = 0.07), there was some indication that Spanish speakers may have responded more favorably to ConTxt plus health-coaching calls than English speakers did (Spanish contrast: −7.90%, *p* < 0.001; English contrast: −1.82%, *p* = 0.107).

Table 4 shows the estimated marginal means, between-group differences, and corresponding 95% CIs for secondary outcomes at each time point. Differences in absolute weight, BMI, and body fat percentage followed a similar pattern as with percent weight loss. Specifically, the group receiving ConTxt plus health-coaching calls achieved significantly greater reductions in each secondary outcome when compared to the group that received standard print materials, but not when compared to the group that received ConTxt only.

**Table 4:** Estimated marginal means, between-group differences, and 95% CIs for the comparison of absolute weight (kg), change in BMI, and change in percent body fat between ConTxt study groups over 12 months.

**Absolute weight change**				
	Standard print versus ConTxt only		Between-group difference	*p*-Value
6 months	−1.25 (−2.52 to 0.03)	−2.12 (−3.47 to 0.88)	−0.93 (−2.76 to 0.90)	0.32
12 months	−0.73 (−2.02 to 0.57)	−1.68 (−2.99 to −0.37)	−0.95 (−2.81 to 0.90)	0.31
	Standard print versus ConTxt plus health-coaching calls			
6 months	−1.25 (−2.52 to 0.03)	−2.85 (−4.18 to −1.52)	−1.60 (−3.44 to 0.24)	0.09
12 months	−0.73 (−2.02 to 0.57)	−3.30 (−4.63 to −1.97)	−2.58 (−4.42 to −0.73)	0.01
	ConTxt only versus ConTxt plus health-coaching calls			
6 months	−2.18 (−3.47 to −0.88)	−2.85 (−4.18 to −1.52)	−0.67 (−2.55 to 1.20)	0.48
12 months	−1.68 (−2.99 to −0.37)	−3.30 (−4.63 to −1.97)	−1.62 (−3.50 to 0.26)	0.09
**BMI change**				
	Standard print versus ConTxt only		95% CI	*p*-Value
6 months	−0.31 (−0.73 to 0.11)	−0.51 (−0.94 to −0.09)	−0.21 (−0.81 to 0.39)	0.50
12 months	0.07 (−0.48 to 0.61)	−0.34 (−0.89 to 0.21)	−0.41 (−1.19 to 0.37)	0.30
	Standard print versus ConTxt plus health-coaching calls			
6 months	−0.31 (−0.73 to 0.11)	−.91 (−1.35 to −0.47)	−0.60 (−1.21 to −.001)	0.05
12 months	0.07 (−0.48 to 0.61)	−.77 (−1.33 to −0.21)	−0.84 (−1.62 to −0.6)	0.04
	ConTxt only versus ConTxt plus health-coaching calls			
6 months	−0.51 (−0.94 to −0.09)	−.91 (−1.35 to −0.47)	−0.40 (−1.01 to 0.22)	0.20
12 months	−0.34 (−0.89 to 0.21)	−.77 (−1.33 to −0.21)	−0.43 (−1.22 to 0.36)	0.29
**Body fat percentage change**				
	Standard print versus ConTxt only		95% CI	*p*-Value
12 months	0.18 (−0.64 to 1.00)	−0.44 (−1.26 to 0.39)	−0.62 (−1.78 to 0.54)	0.30
	Standard print versus ConTxt plus health-coaching calls			
12 months	0.18 (−0.64 to 1.00)	−1.56 (−2.37 to −0.71)	−1.72 (−2.89 to −0.55)	0.00
	ConTxt only versus ConTxt plus health-coaching calls			
12 months	−0.44 (−1.26 to 0.39)	−1.56 (−2.37 to −0.71)	−1.10 (−2.27 to 0.07)	0.07

The model for change in BMI did not adjust for baseline BMI. A linear regression model was performed for body fat percentage adjusted for baseline body fat percentage, age, and gender, because we did not assess body fat percentage at month 6.

Abbreviation: BMI, body mass index; CI, confidence interval

Among those who received the ConTxt interventions, the median (interquartile range) of mean percent of daily engagement declined slightly over time: 28.69 (22.92 to 36.25) at M6 and 24.91 (18.63 to 31.95) at M12. However, a unit increase in mean percent of daily engagement throughout the study was associated with greater percent weight loss (−0.08%, *p* < 0.05).

Adverse events were collected; however, none occurred related to participation in the study.

## Discussion

An automated system that offered 2–4 personalized, tailored, and interactive SMS per day containing content that was theory- and evidence-based and focused on reducing energy intake and increasing energy expenditure was only efficacious when combined with brief monthly phone calls with a health coach. On average, the combined intervention resulted in a weight loss equal to 3.6%, contrasted against those in the control who lost 0.6%, at 1 year among a diverse sample of English- and Spanish-speaking adults who were overweight or obese. The intervention effects were consistent across other weight-related outcomes, such as BMI and body fat percentage measured via DXA, and this finding is consistent with a similar study [13] and contributes further evidence that SMS coupled with brief monthly health coach calls can support sustained weight loss. In contrast, SMS alone does not appear to have a significantly greater effect on weight loss than printed materials. This is aligned with previous research that indicates that SMS content may need to be supported with other methods in order to maximize its efficacy for changing weight-related behaviors [26,27].

The primary finding does not appear to be influenced by age, sex, or ethnicity. However, there was some indication that those who received the intervention in Spanish may have responded more favorably to the intervention than those who received it in English. Given that this finding was generated by prespecified subgroup analyses that may have a high false-positive rate, it should be taken with caution and warrants further investigation. More specifically, future research should examine the extent to which acculturation among Hispanics might influence the efficacy of technology centric weight loss interventions. The present study represents a much-needed addition to the limited existing research on the effects of digital health interventions for behavior change among hard-to-reach populations [28].

Notably, there was only a small decline in engagement over the year-long intervention, and higher engagement, defined as interaction with the SMS content, was associated with increased weight loss. Consensus around how to define and measure engagement within digital health interventions is evolving [29], and our approach is a reasonable one, particularly given that the intervention centered on a high degree of interactivity with the SMS system. However, more research is needed to establish the definitions and metrics that are most useful to understanding intervention effects. Importantly, the heterogeneity of digital health interventions may present a barrier to achieving common metrics of engagement, but standardized qualitative assessments may be well-suited to this task.

The strengths of this study include the recruitment of a diverse sample of English- and Spanish-speaking adults, a randomized design, and a high rate of study completion (84.9%) over 1 year. Those who measured participants and conducted the statistical analyses were unaware of participants’ allocation throughout the study. Additionally, given that the intervention was delivered via an automated SMS system and brief phone calls from a health coach, it encompasses a highly scalable approach to the delivery of theory- and evidence-based weight loss content. The study also has limitations. First, the average weight loss within the group that received health coach phone calls was modest, and although modest amounts of weight loss are likely to be important in the context of a very widely delivered intervention [30], the amount of weight loss may not be sufficient to substantially reduce the risk of debilitating chronic diseases, such as type 2 diabetes [31–33]. Another limitation is that some study staff and health coaches (both English- and Spanish-speaking) who delivered the interventions were not blinded to participant condition. Lastly, this was a single-site study in which the entire sample resided in San Diego County and were primarily (77%) female, which may limit the generalizability of the findings to those in other regions in the USA and males.

### Conclusions

A 1-year intervention that delivered theory- and evidence-based weight loss content via daily personalized, tailored, and interactive SMS was only effective when combined with brief, monthly phone calls. Additional research is needed to determine if this approach is cost-effective, particularly among hard-to-reach populations such as Hispanics, who are disproportionately affected by the negative consequences of overweight and obesity.

## Supporting information

S1 CONSORT Checklist (DOC)

S1 Call Script – English (DOCX)

S1 Call Script – Spanish (DOCX)

S1 Research Plan (PDF)

## Abbreviations

BMI: body mass index
CI: confidence interval
DXA: dual-energy x-ray absorptiometry
M12: month 12
M6: month 6
PAR-Q: Physical Activity Readiness Questionnaire
SMS: short message service
SWM: Strategies for Weight Management
