# Supplementary material for: Text messaging and brief phone calls for weight loss in overweight and obese English- and Spanish-speaking adults: A 1-year, parallel-group, randomized controlled trial
Source: PLoS Med. 2019 Sep 25;16(9):e1002917. doi: 10.1371/journal.pmed.1002917 (PMC6760774; doi:10.1371/journal.pmed.1002917)
Supplement: S1 Call Script – English — (DOCX) [file pmed.1002917.s002.docx]

**ConTxt RCT**

**Counseling Call Script**

**Note:** this is meant to be a guide to help coaches with the structure of counseling calls; however the plan and recommendations provided to participants are the most important component.

**START TIME (*enter the time that you started on this section)*: ___________________ AM PM**

“Hello ______________________. This is ______________, your Health Coach from the ConTxt study. Do you have 10 – 15 minutes to complete your call right now?”

*If yes, continue with script.*

*If no:* “when would be a good time to call you again. The call will take about 10 – 15 minutes.”

*For first time calls only:*

“As outlined during your orientation session at your first appointment, I will be calling you on a monthly basis, twice this first month, over the course of the 12 month study. The purpose of the phone calls are to check in and see how you are doing with your weight loss goals. We’ll talk about any barriers that may have gotten in your way and work together to make a plan to get past those barriers.”

“You’ve now been in the program for ____ week(s). Are you having any technical difficulties with the text messages?”

*If yes, address technical difficulties and send any SMS issues to Mark and CC Angelica and Lindsay.*

*If no, continue with script.*

1. “I’d like to talk to you about your weight loss since our last call (or since starting the program for first time calls). Based on your last reported weight, it looks like you have gained/lost/stayed the same since last time. *(NOTE: If participant is NOT reporting weight via SMS messages, ASK for current weight and compare to last reported weight which may be the baseline weight).*

CURRENT WEIGHT:________________

*If stayed the same or gained weight: Continue onto Q2*

*If lost weight, skip to page -Q11*

1. “What kinds of things make it hard for you to stick to your weight loss plan?”

*Based on response, go to PA Barriers or Calorie Restriction Barriers below. Note if participant mentions both PA and calories restriction as barriers then ask participant:*

“Which habits would you say you are having the most difficulty with? Is it with being more active or with reducing the amount of calories you eat?”

**PA Barriers**

1. “Let’s talk some more about your exercise habits and goals. I’d like to get some more information as to what barriers or roadblocks may have gotten in your way of exercise. What type of activities have you been doing recently?” *(Prompt: How many times a weeks? How long each time?) NOTE: Check the CMS activity log to see if the participant is reporting daily step counts.*
2. “How many steps are you averaging per day on your pedometer? What kinds of things make it hard for you to exercise on a regular basis/or increase your step count by 10% each week [Remind that goal is to reach 12,000 steps every day]?”

For next question(s) refer to call tip sheets for guidance:

1. “Okay, [*list reasons i.e. work, time, etc](_______________________________________________________________________________)* has/have been making time for exercise hard for you. Can you think of any solutions or adjustments in your schedule that you could make?” *Prompt: what about during your lunch time or work breaks? Before or after work? Doubling errands by adding walking or cycling? Doing an exercise DVD at home?*
2. “From the things we just discussed, which things would you say you can commit to doing this week to increase your physical activity/step count/exercise?”_______________________________________________________________________

“Great. To increase your physical activity or exercise level, you will…..”*Prompt: frequency, duration*

1. *If not willing or able to 🡪* “It is important to come up with solutions that will work for you and that you are willing or able to do. What other solutions can you come up with that you think you can commit now to get you started?” *(Repeat steps above)*

“Great! So you will work on [list: ____________________________________________________] starting this week and we can discuss your progress the time we talk on [Date of next call]. How do you feel about committing to this? Do you have any questions before we end today’s call?”

“Thank you [name]! Have a great week! Remember, you can always give me a call if you have any questions. Otherwise, I’ll talk to you again in _________ weeks on [DATE].”

**Calorie Reduction Barriers**

1. “Let’s talk a little bit more about your eating habits. I’d like to get some more information as to what barriers or roadblocks may have gotten in your way of reducing your calories. What kinds of things make it hard for you to reduce your calories every day?” *(Prompt: work schedule, family, portions, don’t plan ahead, money, temptations, etc).*

For next question(s) refer to call tip sheets for guidance:

1. “Can you think of any solutions or ways to reduce your daily calories?” *Prompt: reduce portions, reduce snacking, change cooking methods, use ingredient substitution, talk to the cook of the house, keep track of foods with food log, etc.*
2. From the things we just discussed, which things would you say you can commit to starting in order to decrease your caloric intake?

*Great. To reduce your daily caloric intake, you will…..*

*If not willing or able to 🡪 It is important to come up with solutions that will work for you and that you are willing or able to do. What other solutions can you come up with that you think you can commit now to get you started? (Repeat steps above)*

Great! So you will work on [list:____________________________________________________] starting this week and we can discuss your progress the time we talk on [Date]. How do you fill about committing to this? Do you have any questions before we end today’s call?

Thank you [name]! Have a great week! Remember, you can always give me a call if you have any questions. Otherwise, I’ll talk to you again in _________ weeks.

*If participant has lost weight:*

1. It is great that you have lost weight. Congratulations! Are there any barriers or issues that you think may get in your way this week or that you would like to discuss? *(Probe: any plans to eat out? Be social with friends? Social pressures? Business trips? Motivation issues?)*
2. [*If there are barriers]* what do you think are some potential solutions for [list:___________]?
3. From the things we just discussed, which can you commit to starting this week to decrease your caloric intake or increase your physical activity/steps/exercise?

“Great. To reduce your daily caloric intake/increase your exercise, you will…..”

*If not willing or able to🡪 “*It is important to come up with solutions that will work for you and that you are willing or able to do. What other solutions can you come up with?” *(Repeat steps above)*

“Great! So you will work on [list: ____________________________________________________] starting this week and we can discuss your progress the time we talk on [Date]. How do you feel about committing to this? Do you have any questions before we end today’s call?”

“Thank you [name]! Have a great week! Remember, you can always give me a call if you have any questions. Otherwise, I’ll talk to you again in _________ weeks on [DATE]”.

**END TIME (*enter the time that you finished this section)*: ___________________ AM PM**
