# Supplementary material for: Text messaging and brief phone calls for weight loss in overweight and obese English- and Spanish-speaking adults: A 1-year, parallel-group, randomized controlled trial
Source: PLoS Med. 2019 Sep 25;16(9):e1002917. doi: 10.1371/journal.pmed.1002917 (PMC6760774; doi:10.1371/journal.pmed.1002917)
Supplement: S1 Call Script – Spanish — (DOCX) [file pmed.1002917.s003.docx]

**ConTxt RCT**

**Counseling Call Script-SPANISH**

**Note:** this is meant to be a guide to help coaches with the structure of counseling calls; however the plan and recommendations provided to participants are the most important component.

**START TIME (*enter the time that you started on this section)*: ___________________ AM PM**

“Hola ______________________. Le habla ______________, su consultora de salud del estudio ConTxt. Tendrá disponible entre 10 – 15 minutos para completar su llamada del estudio en este momento?”

*If yes, continue with script.*

*If no:* “cuando será un buen momento para llamarle de nuevo. La llamada tomará entre 10 – 15 minutos.”

*For first time calls only:*

“Como se le informó en la sesión de orientación en su primera visita, le estaré llamando una vez al mes, dos veces en el primer mes, durante el curso del estudio de 12 meses. El propósito de las llamadas telefónicas es ver cómo va en sus metas para bajar de peso. Hablaremos sobre cualquier obstáculo que tenga en su camino y trabajaremos en hacer un plan para vencer esos obstáculos.”

“Usted ha estado en este programa por ____ semanas(s). ¿Ha tenido algún problema técnico con los mensajes de texto?

*If yes, address technical difficulties and send any SMS issues to Mark and CC Angelica and Lindsay.*

*If yes, continue with script.*

1. “Me gustaría hablar con usted sobre su pérdida de peso desde nuestra última llamada (o desde que empezó en programa para la primera llamada). En base a su último peso reportado, parece que usted ha ganado/perdido/se ha mantenido desde la última vez. *(NOTA: Si el participante NO está reportando peso via SMS mensajes de texto, PREGUNTE por el peso actual y compare con el último peso reportado que puede ser el peso de la visita 1).*

PESO ACTUAL:________________

*If stayed the same or gained weight: Continue onto Q2*

*If lost weight, skip to page -Q11*

1. “¿Qué clase de cosas hacen difícil para usted mantenerse en su plan para bajar de peso?”

*Based on response, go to PA Barriers or Calorie Restriction Barriers below. Note if participant mentions both PA and calories restriction as barriers then ask participant:*

“¿Cuáles hábitos diría que usted está teniendo más problemas con? ¿Es con siendo más activo o con reducir la cantidad de calorías que usted come?

**PA Barriers/Obstáculos de Actividad Física**

1. “Hablemos más sobre sus metas y hábitos de ejercicio. Me gustaría tener más información sobre cuales barreras u obstáculos ha tenido que le han impedido hacer ejercicio. ¿Qué tipo de actividades ha estado hacienda recientemente? *(prompt: ¿Cuántas veces a la semana? ¿Cuánto tiempo en cada una de esas veces?) NOTE: Check the CMS activity log to see if the participant is reporting daily step counts.*
2. “¿Cuántos pasos en promedio marca usted por día en su podómetro? ¿Qué clase de cosas hacen difícil para usted que pueda hacer ejercicio regularmente/o que incremente el número de pasos por un 10% cada semana [Recordar que la meta es alcanzar los 12, 000 pasos por día]?”

For next question(s) refer to call tip sheets for guidance:

1. “Okay, [enlister razones, por ejemplo trabajo, tiempo, etc*](_______________________________________________________________________________)* ha hecho que el tiempo para que se ejercite sea difícil para usted. ¿Podría pensar en alguna solución o ajustes en su horario que usted pueda hacer?” *Prompt: ¿Que tal durante su hora del almuerzo o mientras descansa en su trabajo? ¿Antes o después de trabajar? ¿Qué tal doblando diligencias al caminar o andar en bicicleta y hacer sus mandados al mismo tiempo? ¿Siguiendo una rutina de un de DVD de ejercicio en casa?*
2. “De las cosas que acabamos de mencionar, ¿cuáles usted se podría comprometer a hacer durante esta semana para incrementar su actividad física/número de pasos/ tiempo de ejercicio?”_______________________________________________________________________

“Muy bien. Para incrementar su actividad física o nivel de ejercicio, usted hará….”

*Suggest: frequencia, duración*

1. *If not willing or able to 🡪* “Es importante que encuentre soluciones que le servirán y que esté dispuesto o pueda hacerlo. ¿Qué otras soluciones encuentra que piensa que usted pueda cumplir ahora para poder empezar? ” *(Repeat steps above)*

“¡Muy bien! Entonces usted trabajará en [list: ___________________________________________________] empezando esta semana y podremos discutir su progreso la siguiente vez que hablemos el [fecha de la próxima llamada]. ¿Cómo se siente comprometiéndose a ésto? ¿Tiene alguna pregunta antes de que terminemos con la llamada de hoy?”

“¡Muchas gracias [nombre]! ¡Que tenga una excelente semana! Recuerde que puede llamar siempre que tenga una pregunta. De otra manera, hablaré con usted nuevamente en _________ semanas en [FECHA].”

**Calorie Reduction Barriers/**

**Barreras de Reducción de Calorías**

1. “Hablemos un poco más de sus hábitos alimenticios. Me gustaría tener más información sobre que barreras u obstáculos le evitaron poder reducir sus calorías. Qué tipo de cosas hacen difícil que reduzca su consume diario de calorías?” *(Prompt: horario de trabajo, familia, porciones, no planeó con tiempo, dinero, tentaciones, etc).*

For next question(s) refer to call tip sheets for guidance:

1. “¿Puede pensar en cualquier solución o manera para reducir su consumo diario de calorías?” *Prompt: reducir porciones, reducir botanas, cambiar los métodos de cocinar, usar ingredientes substitutos, hablar con el cocinero de la casa, mantener un seguimiento de la comida con el registro de alimentos, etc.*
2. De las cosas que acabamos de mencionar, a que cosas usted diría que se puede comprometer a empezar para disminuir su consume calórico?

*Muy bien. Para reducir su consumo diario de calorías, usted hará….*

*If not willing or able to 🡪* “Es importante que encuentre soluciones que le servirán y que esté dispuesto o pueda hacerlo. ¿Qué otras soluciones encuentra que piensa que usted pueda cumplir ahora para poder empezar? ” *(Repeat steps above)*

¡Muy bien! Entonces usted trabajará en [list:____________________________________________________] empezando esta semana y podremos discutir su progreso la siguiente vez que hablemos el [Fecha]. ¿Cómo se siente comprometiéndose a esto? ¿Tiene alguna pregunta antes de que términos la llamada de hoy?

¡Gracias [nombre]! ¡Que tenga una gran semana! Recuerde que puede llamar cuando tenga cualquier pregunta. De otra manera, hablaré con usted de nuevo en _________ semanas.

*Si el participante ha perdido peso:*

1. Qué bien que haya perdido peso. ¡Felicidades! ¿Existe cualquier barrera u obstáculo que usted piense que pueda afectarle en esta semana o que usted quiera discutir? *(Prompt: ¿algún plan para comer fuera? ¿reuniones sociales con sus amigos? ¿Eventos sociales? ¿Viajes de negocio? ¿Problemas con motivarse?)*
2. [*If there are barriers]* ¿cuáles piensa que son posibles soluciones para [list:___________]?
3. De las cosas que acabamos de mencionar, a cuales se puede comprometer a empezar esta semana para disminuir su consumo calórico o para incrementar su activada física/pasos/tiempo de ejercicio?

“Muy bien. Para reducir su consume diario de calorías/incrementar su ejercicio,

usted hará….”

*If not willing or able to🡪* “Es importante que encuentre soluciones que le servirán y que esté dispuesto o pueda hacerlo. ¿Qué otras soluciones encuentra que piensa que usted pueda cumplir ahora para poder empezar? ” *(Repeat steps above)*

“¡Muy bien! Entonces usted trabajará en [list: ___________________________________________________] empezando esta semana y podremos discutir su progreso la siguiente vez que hablemos en [Fecha]. ¿Cómo se siente comprometiéndose a esto? ¿Tiene alguna pregunta antes de que terminemos su llamada de hoy?”

“¡Gracias, [nombre]! ¡Que tenga una excelente semana! Recuerde que me puede llamar cuando sea si tiene alguna pregunta . De otra manera, hablaré con usted de nuevo en _________ semanas el [FECHA]”.

**END TIME (*enter the time that you finished this section)*: ___________________ AM PM**
