# Supplementary material for: Text messaging and brief phone calls for weight loss in overweight and obese English- and Spanish-speaking adults: A 1-year, parallel-group, randomized controlled trial
Source: PLoS Med. 2019 Sep 25;16(9):e1002917. doi: 10.1371/journal.pmed.1002917 (PMC6760774; doi:10.1371/journal.pmed.1002917)
Supplement: S1 Research Plan — (PDF) [file pmed.1002917.s004.pdf]

UCSD Human Research Protections Program
New Biomedical Application
RESEARCH PLAN

Instructions for completing the Research Plan are available on the HRPP website.
The headings on this set of instructions correspond to the headings of the Research Plan.
General Instructions: Enter a response for all topic headings.
Enter "Not Applicable" rather than leaving an item blank if the item does not apply to this project.

Version date: 9/30/2013

1. PROJECT TITLE

ConTxt: A Text Message Intervention for Weight Loss (data analysis)

2. PRINCIPAL INVESTIGATOR

Kevin Patrick, MD, MS

3. FACILITIES

University of California, San Diego
Atkinson Hall, room 3501

4. ESTIMATED DURATION OF THE STUDY

The estimated duration of the study is two years (August 2018 – August 2020).

5. LAY LANGUAGE SUMMARY OR SYNOPSIS (no more than one paragraph)

This is a continuation of protocol # 091040 which expires in 2018. The purpose of this protocol is to complete data analysis and prepare manuscripts for publication. The ConTxt study was a 4-year randomized controlled trial funded by the National Cancer Institute to develop and test the efficacy of a text message intervention for weight loss among overweight and moderately obese (BMI 27–39.9) English and Spanish speaking men and women ages 21–60. The ConTxt study's aims were to expand previous work by customizing participant's text messages based on their location and environmental influences related to food and level of physical activity. Text messages targeted both English and Spanish speaking participants. The ConTxt study randomized 298 participants for a 12 month program. Participants were randomly divided into one of three groups: text messages only, text messages plus monthly counseling calls, or a control comparison group with only printed materials.

6. SPECIFIC AIMS

The **primary aim** of this study will be to evaluate differences from baseline in percent weight loss at 12 months. We hypothesize that, compared to the usual care Standard Print condition differences in weight loss will be significant in both the **SMS only (SMS = short message service or text message)** and **SMS+Phone** intervention groups. We will also determine differences among groups in BMI and weight in kg.

The **secondary aims** will be to assess, at 6 and 12 months, treatment effects on:

- The proportion of each study group that loses at least 5% of initial body weight
- Diet, physical activity and sedentary behaviors, quality of life and depression
- Level of satisfaction and use of intervention components (e.g. SMS and phone counseling)
- Differences in percent weight loss between the **SMS only** and **SMS+phone** intervention groups.

Exploratory Analyses will include study of:

- Effect of "dose" of intervention on outcomes at 6 and 12 months
- Examination of mediators of intervention effects (e.g., psychosocial constructs, eating behaviors, physical activity, sedentary behaviors, changes in home environment)
- Examination of moderators of intervention effects (e.g., sex, age, education level, neighborhood characteristics)
- Treatment effects among those who prefer the Spanish language versions of ConTxt.
- Examination of patterns of physical activity using a Global Positioning System (GPS) device. A 25% sub-sample of participants (n=78) received a GPS device to wear for seven days at their baseline and 12-

month measurement appointments. Collection of both location and accelerometer data allowed us to examine patterns of physical activity using our developed Physical Activity and Location Measurement System (PALMS) among participants in a weight loss study. PALMS is comprised of an integrated suite of hardware, software and database solutions that supports capture and subsequent analyses of data on PA from a geospatial perspective. Data analysis from PALMS will occur now that study participants have completed the ConTxt research study.

7. BACKGROUND AND SIGNIFICANCE

Overweight/Obesity & Health Overweight and obesity are prevalent conditions in the United States. In 2003-2004, 17.1% of US children and adolescents were overweight and 32.2% of adults were obese. In addition, there were significant differences in obesity prevalence in racial and ethnic groups. In 2003-2004, approximately 30% of non-Hispanic white adults were obese as were 45% of non-Hispanic black adults and 36.8% of Mexican Americans [13]. Overweight and obesity are associated with increased risk for many diseases. In particular, visceral obesity, increases an individual's risk of morbidity from hypertension, dyslipidemia, type 2 diabetes mellitus, coronary heart disease, stroke, gallbladder disease, osteoarthritis, and certain types of cancer. Obesity has been associated with increased cancer risk. In particular, obesity has been associated with increased incidence of cancer of the gallbladder, prostate, pancreas, stomach, colon, endometrium, ovary, kidney, esophagus, and breast. In addition, men and women with a lifetime maximum body mass index meeting obesity criteria (BMI>30 kg/m2) are at greater risk of developing non-Hodgkin's lymphoma.

Research has shown that weight reduction in obese individuals with hypertension, dyslipidemia, and type 2 diabetes, even in modest (5-10%) amounts, results in a variety of health benefits, and is considered the most effective non-pharmacologic method of improving health status. Decreasing the overall incidence of overweight, obesity, and obesity-related health conditions, as well as the existing racial and ethnic disparities in obesity prevalence, is a major public health concern. It has been known for over 30 years that a 10% reduction in weight in men corresponds to an approximate 20% reduction in coronary disease incidence, whereas a 10% increase in weight is associated with a 30% increase in cardiovascular morbidity. In 2001, an estimate of the economic burden of overweight and obesity totaled $117 billion, including direct and indirect costs associated with heart disease ($8.8 billion), type 2 diabetes ($98 billion), hypertension ($4.1 billion), osteoarthritis ($21.2 billion), gall bladder disease ($3.4 billion), breast ($2.9 billion), endometrial ($933 million), and colon ($3.5 billion) cancers.

Dietary Behaviors Poor dietary behaviors are a known risk factor for the development of obesity, as well as for diabetes, CHD, cancer and stroke. Research supports that a diet rich in fruits and vegetables and low in fat is important in preventing these chronic diseases, and is recommended by the USDA, USDHHS, Surgeon General, NRC, NHLBI, NCI, ACS, and AHA. Although national surveys indicate a decline in the average proportion of calories from total and saturated fat over the past several decades, the CDC estimated in 2000 that only 38% of individuals 2 years and older met the recommendation for total fat intake and 41% of these individuals met the recommendation for saturated fat intake. In addition, data from the 2005 Behavioral Risk Factor Surveillance System (BRFSS) showed that only 32.6% of the U.S. adults consumed fruit two or more times per day, and only 27.2% ate vegetables three or more times per day. Simple dietary restriction has not been associated with successful weight control and may result in a nutritionally inadequate diet. Thus, rather than focusing only on limiting total energy intake, it is important to promote a diet that is nutrient dense: high in vegetables, fruits, grains, and other fiber-rich plant foods, yet low in fat, at a given level of energy intake.

Physical Activity (PA) reduces the risk of developing several leading chronic diseases including obesity, coronary heart disease, and type 2 diabetes. Research shows that persons who are overweight or obese but physically active have much lower death rates from cardiovascular disease and all-cause mortality than people who are sedentary and unfit. Yet half of US adults do not meet current PA guidelines. In 2005, only 46.7% of women and 49.7% of men reported engaging in regular leisure-time physical activity. Research and national guidelines suggest that PA is an integral component of weight loss interventions as it contributes moderately to

weight loss, helps with weight loss maintenance, and reduces the increased health risks associated with overweight and obesity. PA can help prevent and control obesity through several mechanisms. Exercise increases energy expenditure, reduces abdominal and visceral fat, builds lean body mass, and moderately increases metabolic rate.

Mobile Interventions for Health Behaviors
Current mobile phone platforms vary with respect to operating systems and include systems such as Microsoft Windows Mobile, Symbian, Blackberry, Palm OS, Mobile Linux, J2ME, and the Android platform recently announced by Google. Software used to program applications in these environments differs and programs developed to run on one do not run on others. While this raises important policy issues for interoperability of health applications, there is one communication approach (other than voice) that operates on essentially all mobile phones: short message services (SMS) or text messages. Length of present day SMS messages is in the 140 to 160 character range, roughly the length of a short sentence or two. Text messages sent to and from mobile phones will more than double over the next five years to 2.3 trillion messages by 2010, according to a Gartner survey (www.gartner.com). Some 936 billion SMS (short message service) messages were transmitted in 2005 and, while use is more common among younger individuals, use among adults is increasing. A Pew/Internet survey released in 2008 (Pewinternet.org) found 58% of American adults who owned cell phones reporting use of SMS messages.

While the computing power and portability of mobile phones make possible new applications for automatic, timely, and tailored presentation of health messages, a review of the literature shows limited research on mobile applications. Several studies have successfully used mobile phones and PDAs for assessment and data collection in clinical trials. The Wireless Health Outcomes Monitoring System (WHOMS) project, achieved 100% completion rate for the cancer patients who used mobile phones to answer quality of life questionnaires and report symptom changes. PDAs were used in a study of the relationship between mood and binge eating and cell phones were used in a study of mood and drinking behavior.

SMS messaging is beginning to show evidence of efficacy in helping individuals adopt and maintain important health-related behaviors. For example sending text messages to mobile phones increased the effectiveness of a smoking cessation intervention among college students. Another study among young adults in New Zealand revealed that participants who received text messages were more likely to quit smoking at 6 weeks compared to controls. In a program conducted among youth with type 1 diabetes daily text messages were helpful for disease self-management, increased self-efficacy and treatment adherence and achieved high satisfaction among participants. In a randomized controlled trial of an Internet and mobile phone–based physical activity (PA) intervention among overweight adults that included reminders for exercise sessions sent via cell phone, experimental participants engaged in over 2 hours more PA per week than those with no access. In an uncontrolled study among adults in Korea, sending text messages once a week about diet, exercise and behavior modification proved effective in promoting weight loss at 12 weeks. <u>To date, there have been no studies on the use of **daily** text messages sent to and received from mobile phones as an intervention to address overweight/obesity among adults.</u> <u>Further, very few of these studies have explored the theoretical basis of these interventions.</u>

Need for Research on the Theoretically Derived SMS-based Health Behavior Interventions
The prevention and treatment of obesity is proving to be one of the most important public health issues of our times. While it's <u>un</u>likely that SMS messaging will be <u>**the**</u> solution to this problem, the frequency and simplicity of SMS messaging to and from one's mobile phone to prompt behaviors such as self-monitoring of weight, pedometer step counts, eating behaviors, and healthy food choices might contribute meaningfully to the armamentarium of treatment options. The weight loss literature clearly demonstrates the superiority of interventions that are theoretically derived. The majority of them apply Cognitive-Behavioral strategies derived from Social Cognitive Theory. Examining effective approaches to weight loss may be helpful for

determining what behavioral skills should be promoted for weight loss and weight loss maintenance. Among these, self-monitoring has emerged as a critical skill for obesity management as those who report monitoring their weight on a daily or weekly basis have greater success in achieving weight loss goals. Self-monitoring increases awareness of food and caloric intake, enhances self-efficacy, and allows for monitoring of progress and change over time. However, detailed self-monitoring is labor-intensive for participants and adherence is relatively low. One weight loss study found that while self-monitoring of food intake was rated as a useful strategy for weight loss, only 30% of participants continued this behavior after the study had ended. Barriers such as stress, lack time and social support, and discomfort with recording can affect adherence to self-monitoring.

8. PROGRESS REPORT

Formative Research: Pilot Testing: We developed an 8-week pilot test to test 8-weeks of our intervention of text messages, printed materials, and counseling call scripts. We enrolled 20 study participants (12 females, 8 males; 15 Latinos) into the study in May 2011. During the 8-week pilot study, participants completed baseline surveys consisting of demographic items, the Short Acculturation Survey for Hispanics (SASH; for those that identified as Latinos), and the Weight Behavior Inventory (35-items on weight management strategies). Participants also had their height and weight evaluated. All study participants received daily text messages and a binder of supplemental materials related to nutrition, physical activity, and behavioral skills on weight loss. In addition, weekly scripted calls were conducted during weeks 2-8 (7 calls completed) to gain feedback on the program and assess individual weight loss progress. These calls included both quantitative (closed-end response options) and qualitative (open-ended responses) and were completed usually within 10-15 minutes (some weeks calls were as short as five minutes and there were few occasions were calls lasted up to 20 minutes). At the end of the 8-week study, participants had their height and weight taken, completed follow-up surveys including the Weight Behavior Inventory and a satisfaction survey and completed an in-depth interview about their experience in the pilot study.

Formative Research: Pilot Testing Results:

Eighteen participants completed all measures. On average, participants lost 1.9 kg ($p < .01$, 95% CIŒî 0.7 to 3.0), decreased BMI by 0.1 kg/m2 ($p<.01$, 95% CIŒî 0.29 to 1.1), and increased WBI scores by 41% (1.5 points)($p < .01$, CIŒî 1.1 to 1.8). At baseline, there were no differences in WBI scores ($p=.48$, CIŒî -0.3 to 5.8) by gender. There were no differences in weight, BMI, or WBI scores by language type. At 8 weeks, there were no differences in WBI scores ($p=.47$, CIŒî -0.7 to 0.3) by gender, but Spanish speakers had higher WBI scores (M=3.8) than English speakers (M=3.3) ($p=.02$, CIŒî -0.96 to 0.08). There were no differences in weight ($p=.95$, CIŒî 19.1 to 20.5) or BMI ($p=.72$, CIŒî -0.5 to 3.8) by language type. Lastly, 94% of participants reported satisfaction with the program and said they would recommend it to a friend. Feedback from the pilot study was used to improve and develop the 12-month intervention to be evaluated in the randomized controlled trial.

Randomized Controlled Trial Recruitment:

Participants were recruited from October 2011 – March 2013 from the community via newspaper ads, flyers, email list servs, announcements on craigslist, community events like farmer's markets, and through word of mouth. Interested individuals called the telephone number provided on the recruitment materials. Bilingual recruitment staff answered the telephone and screened potential participants for meeting the following inclusion criteria: 21– 60 years old, overweight (BMI >= 27 – 39.8), owns a cell phone capable of sending and receiving SMS messages, not taking medications known to cause weight gain, no history of eating disorder or weight loss surgery, and willingness and ability to comply with study protocols. Interested individuals were also screened using the Physical Activity Readiness Questionnaire (PAR-Q) and any individual answering yes to at least one

of the PAR-Q questions needed to have his/her doctor's approval before being eligible to participate. Eligible individuals then scheduled a baseline visit.

Figure 1. Flow diagram of participants

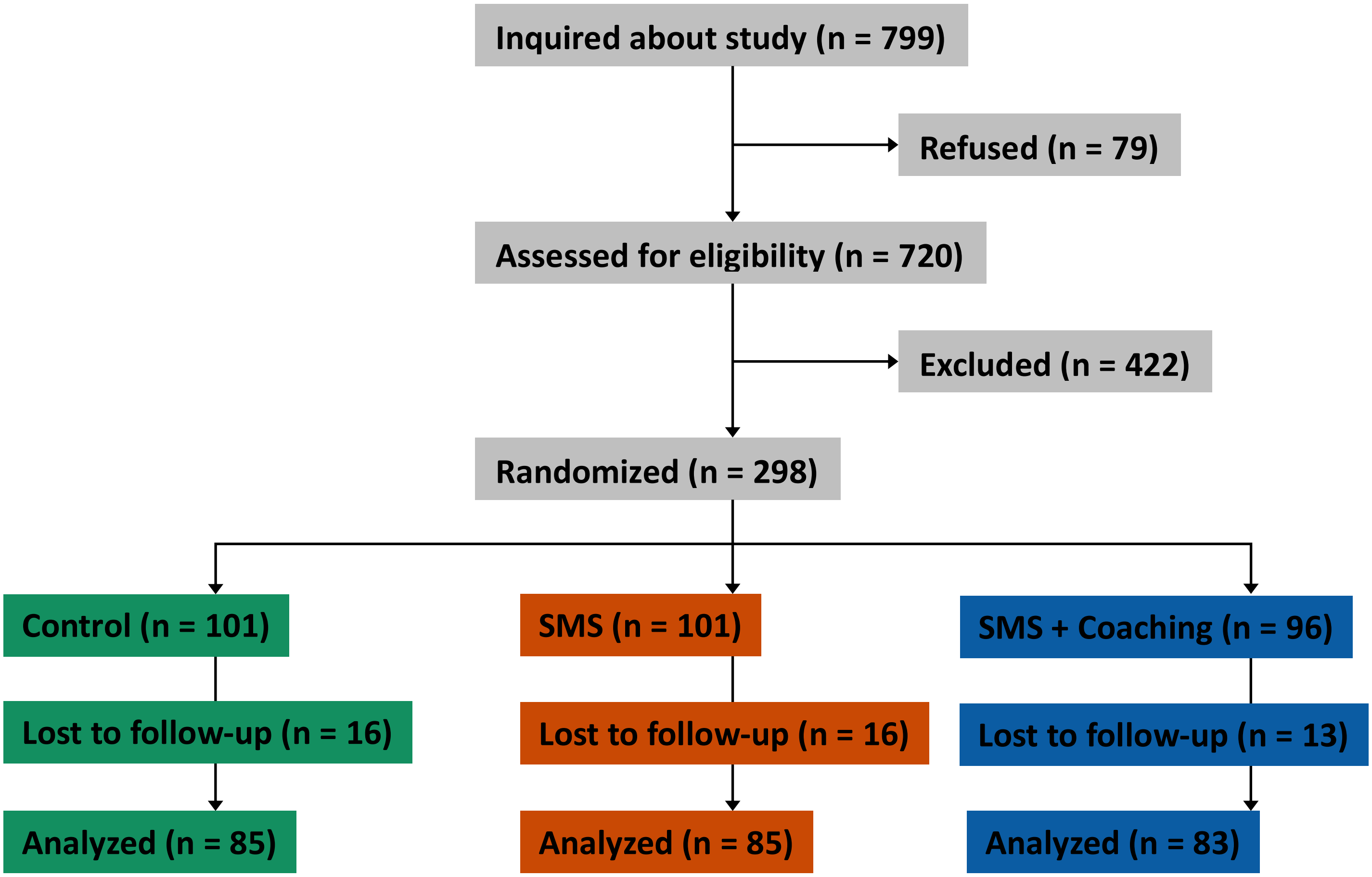

Table 1. Reasons for Ineligibility

Reason	Count
Over 60 years old	14
Under 21 years old	6
BMI over 39.9	85
BMI under 27	72
No Cell Phone/Not able to send/receive SMS or MMS messages	22
History of Eating Disorder	3
Household member/Roommate already participating in the study	5
Medical Condition or medication	33
Eligibility Unknown/Did not return phone calls/Did not complete screener/Flagged-no response from physician	105
Non-SD County Resident	25
Other Reason	6
Participating in Another Weight Loss Program	11

Pregnant	8
Recently quit smoking/Process of trying to quit smoking	2
Taking a weight loss supplement	6
Weight loss surgery	8

Randomized Controlled Trial Measurement:
The following table shows the number of assessment visits completed:

	**Baseline**	**6-months**	**12-months**
Official Drops	0	16	17
Completed Visits	**298**	**261 (87.6%)**	**253 (84.9%)**
Non-compliant	0	21	28
TOTAL	298	298	298

A total of 17 participants were officially removed or withdrawn from the study for the following reasons: became pregnant (n = 3); passed away (n=1); no longer wanted to participate (n=9); moved out of SD County (n=1); did not comply with study protocols (n=2); and deported outside of the US (n=1). The remaining participants that did not complete A2 or A3 assessments were lost to follow up (unable to reach after numerous contact attempts via email, mail, phone, text).

RCT Preliminary results

The following results were presented at the 7th Annual International Society for Research on Internet Interventions 7th Scientific Meeting. October 23 – 25, 2014:

Table 2. Baseline characteristics

**Characteristic**	**Value**
Age (years)*	41.7 (11.1)
% Male	23%
% College graduate or above	44%
% Employed	78%
% Hispanic	41%
% Primarily Spanish speaker	21%
BMI (kg/m^2)*	32.68 (3.39)

*Values are mean (SD)

261 (87.6%) and 253 (84.9%) participants completed the 6 and 12-month visits respectively. Drop out did not differ by treatment condition. Mean (SD) percent weight loss at 12 months was: Control condition-0.47% (6.4%); SMS only-1.99% (8.2%); SMS+Coach-3.54% (8.3%). Compared to Controls, and controlling for BMI at baseline, the SMS only group lost 1.20% more weight (p=.214); and the SMS+Coach lost 2.99% more weight (p=.003). These results illustrate that a 1-year obesity intervention utilizing daily interactive and tailored SMS was most effective when messages were coupled with brief monthly phone coaching.

The following results (Cameron, N., Godino, J., Nichols, J., Wing, D., L., Hill., and Patrick, K. *Associations of Physical Activity, Body Fatness, and Visceral Adiposity in overweight/obese Latino and Non-Latino Adults*) were presented at the 2016 Western Student and Resident Medical Research Forum (January 28 – January 30, 2016):

A total of 236 participants (98 Latinos, 140 non-Latinos) were included in this analysis. Participants had a mean age of 43 years, 22% were male, and mean body mass index was 32.55 kg/m^2. Each measure of Moderate Vigorous Physical Activity [MVPA] (except time per bout) was negatively associated with body fatness, whereas only percentage of time in MVPA and time in non-bouts of MVPA were associated with VAT (all adjusted for age, sex, ethnicity, and randomization and $p < 0.05$). The associations of percentage of time in MVPA and number of bouts with body fatness were stronger in non-Latinos compared to Latinos, but ethnicity did not influence associations with VAT (all adjusted for age, sex, and randomization and significant interaction terms $p < 0.05$).

The following results (Engelberg, J.K., Godino, J., G., Sallis, J., Conway, T.L., Hill, L., and Patrick, K.) *Moderators of the relation between perceived neighborhood safety and physical activity* were presented at the 2016 Society of Behavioral Medicine Meeting (March 30 – April 2, 2016):

Individual demographics moderated the walking safety environment association with total MVPA in 3 interactions (P<.05). Males had more MVPA in neighborhoods with higher walking safety (i.e. 25 min difference high vs. low), with little difference among females. For White non-Hispanics, walking safety was positively related to MVPA, with little difference for Hispanic and other minority participants. Walking safety was positively related to MVPA for overweight participants (20 min difference) and obese Class 1 participants (10 min difference), but not for obese Class 2 participants. The walkability index was moderated by age with a 10 min positive difference in MVPA/day among older participants (51-64 years), an inverse association with MVPA among young adults (23-35 years), and no relation among middle-aged adults (36-50 years).

Publications: The following are our publications and presentations to date on data from this study:

Gonzalez, E., Barrera-Ng, A., Dillon, L., Sullivan, M., Norman, G., & Patrick, K. *A pilot study to evaluate cultural differences between Latinos and Non-Latinos in a text-message based intervention for weight loss.* Presented at the 2011 SACNAS National Conference, October 27-30, 2011 in San Jose, California.

Kolodziejczyk, J.K., Barrera-Ng, A., Patrick, K., Dillon, L., Marshal, S., Arredondo, E., Rock, C., Raab, F., Griswold, W., Sullivan, M., and Norman, G. (2012). Design of a tailored text-message intervention for weight loss. *Circulation*, 125, p386.

Kolodziejczyk, J.K., Barrera-Ng, A., Patrick, K., Dillon, L., Marshal, S., Arredondo, E., Rock, C., Raab, F., Griswold, W., Sullivan, M., and Norman, G. (2012) A text-message based weight loss pilot study for English and Spanish-language speakers. Society of Behavioral Medicine. *Annals of Behavioral Medicine*, 43 (1), s190.

Kolodziejczyk, J.K., Dillon, L., Barrera-Ng, A., Raab, F., Sullivan, M., Griswold, W., Patrick, K., and Norman, G. (2013). An innovative method to tailor text-messages based on user preference in a weight loss randomized controlled trial. Presented May 16, 2013 at the International Society for Research on Internet Interventions Conference, Chicago, IL.

Kolodziejczyk, J.K., Norman, G.J., Barrera-Ng, A., Dillon, L., Marshal, S., Arredondo, E., Rock, C., Raab, F., Griswold, W., Sullivan, M., and Patrick, K. (2013) Feasibility and effectiveness of an automated bilingual text message intervention for weight loss: Pilot study. *Journal of Medical Internet Research Protocols* (2:2), e48.

Kolodziejczyk, J.K., Norman, G.J., Roesch, S., Rock, C., Arredondo, E., Madanat, H., and Patrick, K. Exploratory and confirmatory factor analyses and demographic correlate models of the Strategies for Weight Management (SWM) measure for adults. Presented at the 35th Annual Meeting & Scientific Sessions of the Society of Behavioral Medicine. April 23-26, 2014. Philadelphia PA.

Patrick, K., Raab, F., Rock, C., Norman, G.. Kolodziejck, J., Arredondo, E., Griswold, W., Dillon, L., & Donohue, M. Outcomes of a one-year text message intervention for obese English and Spanish-speaking adults: ConTxt. Presented at the International Society for Research on Internet Interventions 7th Scientific Meeting. October 23 – 25, 2014. Valencia, Spain.

Margaret Crawford, Godino J, Merchant G, Rock C, Marshall S, Arredondo E, Griswold W, Norman G, Donohue M, Kolodziejczk J, Raab F, Dillon L, Patrick K. Impact of a One-Year Text Message Intervention on Physical Activity and Diet in Overweight/ Obese Adults: ConTxt. Poster Presentation at: Society of Behavioral Medicine Conference 2016, Washington, D.C., April 2016.

Margaret Crawford, Godino J, Merchant G, Rock C, Marshall S, Arredondo E, Griswold W, Norman G, Donohue M, Kolodziejczk J, Raab F, Dillon L, Patrick K. Impact of a One-Year Text Message Intervention on Physical Activity and Diet in Overweight/ Obese Adults: ConTxt. Poster Presentation at: UCSD Public Health Research Day 2016, San Diego, CA, April 2016.

Cameron N., Skipper, T., Godino, J., Dillon, L., Waalen, J., Hill, L., and Patrick, K. The Association between changes in depression with changes in body composition over a 12-month period in overweight/obese adults using DXA. Presented at the American College of Physicians Southern California Chapters I, II, and III Poster Competition in Marina del Rey, California on September 16, 2017.

Cameron, N., Godino, J., Nichols, J.F., Wing, D., Hill, L., & Patrick, K. (2017). Associations between physical activity and BMI, body fatness, and visceral adiposity in overweight or obese Latino and non-Latino adults. *International Journal of Obesity, 41*(6), 873-877.

9. RESEARCH DESIGN AND METHODS

This is a continuation of protocol # 091040. The purpose of this protocol is for data analysis and manuscript preparation related to the study activities from protocol 091040. There is no contact (recruitment, consent, enrollment, or data collection) occurring under this new protocol. The study activities from protocol # 091040 that are being analyzed are described below:

Two focus groups with English speakers (5 men, 5 women)

Phase I: Formative Research: Pilot testing the 8-Week ConTxt

We developed an 8-week pilot test to test 8-weeks of our intervention of text messages, printed materials, and counseling call scripts. We enrolled 20 study participants (12 females, 8 males; 15 Latinos) into the study in May 2011. During the 8-week pilot study, participants completed baseline surveys consisting of demographic items, the Short Acculturation Survey for Hispanics (SASH; for those that identified as Latinos), and the Weight Behavior Inventory (35-items on weight management strategies). Participants also had their height and weight evaluated. All study participants received daily text messages and a binder of supplemental materials related to nutrition, physical activity, and behavioral skills on weight loss. In addition, weekly scripted calls were conducted during weeks 2-8 (7 calls completed) to gain feedback on the program and assess individual weight loss progress. These calls included both quantitative (closed-end response options) and qualitative (open-ended responses) and were completed usually within 10-15 minutes (some weeks calls were as short as five minutes and there were few occasions were calls lasted up to 20 minutes). At the end of the 8-week study, participants had their height and weight taken, completed follow-up surveys including the Weight Behavior Inventory and a satisfaction survey and completed an in-depth interview about their experience in the pilot study.

Phase II: Randomized Controlled Trial

298 participants were randomized to one of three groups for a 12-month study period. The 3 groups include:

1. SMS only (text messages only) = Group A
2. SMS (text messages) and phone counseling = Group B
3. Standard Print Materials (Comparison Group) = Group C

Computer Expert System. At baseline, participants completed the Weight Management Strategies Inventory, to identify unique weight loss and weight management strategies for each participant (e.g., self-monitoring, refusing offers of food, eating in response to emotions, exercise behaviors). We also used baseline step counts from the pedometers and self-reported daily bouts of 1000 steps in 10 min to provide the basis for targeted step-count increases at the beginning of the intervention and until participants reached their step-count goals. Responses to each of these assessments were used to develop tailored text messages for each participant. In order to facilitate long-term use and personalization of the program, participants were reassessed at 6 months (half way through the intervention) to update their message content. For example, if a participant succeeded in attaining a behavioral goal the system re-prioritized goals based on an updated assessment and sent new relevant and appropriate messages.

Participants were also able to choose the times that they wanted to receive messages. The pilot system was designed to adapt to user behavior and adjust message frequency based on inferred preferences and this was strengthened in the version tested in this study to provide variability if/when participants wanted it. In addition, participants were able to request modifications to the message schedule at any time. This design maintained a level of interest and utility with the system and reduced the likelihood that a participant was annoyed or 'nagged' by messages, potentially minimizing its effectiveness.

Group A and Group B Intervention Components

The goals of the Group A and Group B intervention included:

- 5% weight loss
- 500 k/cal per day deficit through reduced caloric intake and increased energy expenditure
- 12,000 steps per day through the use of a pedometer

- reduce sedentary behaviors and sitting time

Ongoing intervention components:

1. **Text messaging ("Pull"/"Push" communication)**. The advantage of mobile technology is the ability to contact the user at any time throughout the day and research has shown that increased contact may improve weight loss outcome. Intervention contacts were made through text and picture messaging. Messages were time-based (determined by the participant to be beneficial) or data-based (e.g., in response to something the user entered). Some messages were directly related to the participant's profile (e.g. behavioral strategies that they need to work on based on baseline assessment) and some were general behavior change strategies. "Pull" messages asked the user to enter data. For example, a prompt that asked the participant to enter their mood at a given moment. "Push" messages responded to entered data. For example, after having entered that the user was feeling 'stressed', a message appeared with helpful strategies for stress reduction that are not food-related. Given the novelty of this mode of intervention contact, satisfaction surveys at post-treatment focused on preference for contact frequency and whether these prompts were helpful or not to refine programs for the future.
2. **Food monitoring**. Because self-monitoring of food is so crucial to successful weight management (Wing & Hill, 2001), participants were asked to log their food choices each day on food logs provided in the print materials. Participants logged the time of day, type of food, and amount.
3. **Weekly weighing**. Since weight monitoring has been shown to be an effective tool for weight management (Wing & Hill, 2001), participants were asked to weigh themselves once each week and enter their weight on their phone. Participants were instructed to weigh themselves on the same day and at the same approximate time for consistency.
4. **Steps monitoring**. Based on feedback from our pilot study, participants were sent SMS messages that prompted them to report their daily steps from the previous day. Participants were provided with a list of step conversions for common activities in case they forget to wear their pedometer or if they participated in an activity such as swimming or cycling.
5. **Printed Materials**. Participants were given a ConTxt SMS/MMS Guide and 16-weeks of printed materials at their baseline visit. The 16-weeks of printed materials included weekly topics related to weight loss such as self-monitoring, portion control, and sedentary behavior. The weekly topics also included ConTxt weekly "to do" lists which included a list of suggested challenges related to the weekly topic along with printed food and activity logs. After the first 16 weeks, participants were sent either via regular mail or email "booster" session tip sheets each month for the remaining 8 months of the study. The "booster" session tip sheets were 1-2 pages and included seasonal topics such as Surviving the Holidays or Seasonal Fruits and Vegetables and also behavioral tip sheets such as Overcoming Weight Loss Plateaus.
6. Importantly, we also refined and developed a full Spanish-language version of this based upon the pilot study among Latinos during our Phase I formative research described below and upon feedback from the small pilot study in this population we performed in this study. Text messages each week corresponded to the topics covered by the printed Guide.

Participants randomized to Group B (SMS & Counseling Calls) also received:

7. **Bi-Weekly and Monthly Phone Calls.** Participants received a total of 13 counseling calls their Case Manager assigned at baseline. Two calls were made during the first month in the program and calls were administered monthly in months 2 – 12. This call lasted approximately 15 minutes and allowed for the Case Manager and participant to discuss their progress and problems including barriers and challenges. For Spanish speaking participants, monthly counseling calls were conducted in Spanish by a Spanish speaking Case Manager.

Control Group (Group C)

Participants randomized to the Comparison condition received a binder with an attractive set of Standard Print Materials related to weight loss that is comparable to what one would receive from community resources such as libraries, magazines and national non-profit or governmental organizations such as 5-A Day, American Heart Association and the like. Materials were provided in English or Spanish, as appropriate, and drew upon a large library of previous studies. This included information on diet, sedentary and physical activity behaviors as well as recommendations to move to a healthier weight. In addition, participants received a monthly 1 – 2-page tip sheet on overall health and weight loss related information in the mail or via email. To increase the likelihood that Comparison participants will continue with they study and participate in measurement visits for the full 12 months, after the final measurement visit we offered this group 4-months of the SMS only version of ConTxt at no cost. We did not assess additional outcomes in this group after this post-study 4-month period of use. Our experience with other studies in overweight adults is that incentives like this can be valuable in terms of reducing attrition.

Measurements for ALL Participants

Assessments were conducted for all participants at baseline, 6 and 12 months. Table 5 summarizes measures and collection time points. Survey measures with an asterisk indicate instruments with validated Spanish language versions. We worked to obtain or develop Spanish language translations of additional survey measures.

Table 5: Measurement schedule

**MEASURES**	**BASELINE**	**6-MONTHS**	**12-MONTHS**
PRIMARY OUTCOME			
Body Weight (kilograms)	X	X	X
SECONDARY OUTCOMES			
DEXA (% body fat)	X		X
Diet History Questionnaire 1	X	X	X
Global Physical Activity Questionnaire	X	X	
Actigraph Accelerometer	X		X
Self- report sedentary behavior	X	X	X
EXPLORATORY OUTCOMES			
Eating Behaviors (Weight Management Inventory)	X	X	X
Well Being: SF-12	X		X
Sleep	X	X	X
Self-Esteem	X	X	X
Body Image	X	X	X
Depression	X	X	X
Home Physical Activity Equipment	X		X
Abbreviated Neighborhood Environment Walkability Scale	X		
Psychosocial constructs: self-efficacy, support, changes strategies	X	X	X
Patterns of physical activity: GPS & GIS (*sub-sample only)*	X		X

Demographics	X		
Acculturation Scale	X		
SATISFACTION			
Implementation & Satisfaction	ONGOING		

Physiological Measures

• **Body weight** was measured to the nearest 0.1 kilograms using a calibrated digital scale. Height (without shoes) was measured to the nearest 0.1 cm using a stadiometer with the subject standing erect against a wall with heels close to the wall. **Body mass index (BMI)** was calculated from height and weight as kg/m^2.

- **Body fat percentage** was assessed by dual energy x-ray absorptiometry (DEXA). DEXA provides a whole-body scan measure of body composition as well as regional measurements of fat mass, lean mass, and bone mass. The minimal radiation dose is safe and appropriate for a pediatric population. All scans were conducted by an experienced technician certified by the state of California.

In the event that the DXA technician who is conducting the DXA scan noticed anything unusual on the DXA scan, the following protocol was followed:

- The DXA technician was to follow-up with the radiologist associated with reviewing scans for the DXA machine.
- The radiologist was to be provided a recommendation based on the DXA scan (for example, follow up with the participant's primary care physician for further evaluation).
- The recommendation was to be communicated to the study participant. If the radiologist recommended that the participant follow-up with his/her primary care provider, a copy of the DXA scan was to be mailed to the study participant.
- Receipt of the copy was to be confirmed via phone call from the Coordinator

Behavioral measures of physical activity and diet

• **Diet History Questionnaire (DHQ-1** was the method used to estimate usual dietary intake. The Diet History Questionnaire (DHQ) is a food frequency questionnaire (FFQ) developed by staff at the Risk Factor Monitoring and Methods Branch (RFMMB). This FFQ consists of 124 food items and includes both portion size and dietary supplement questions.

• **The Global Physical Activity Questionnaire (GPAC)** was used to assess physical activity. The GPAQ was developed by the World Health Organization and collects physical activity information in 3 settings: activity at work, travel to and from places, and recreational activities.

• **The Actigraph Acceleromter (model GT3X)** was worn by 30% of the total sample (randomly selected). The GT3X Actigraph (www.theactigraph.com) is a small (3.8 x 3.7 x 1.8 cm), lightweight (27 g), tri-axial accelerometer that is worn on a belt snugly around the waist. The Actigraph has a rechargeable lithium battery with a between charge battery life of 14 days. The Actigraph technology has been shown to be valid for quantifying activity levels in laboratory and field settings (Nichols et al., 2000). Participants were asked to wear the Actigraph for 7 consecutive days. Participants were given verbal and written instructions on how to properly wear the device. Participants were contacted each day via text message and/or email each day to remind them to wear the accelerometer. They were provided an addressed and pre-paid envelope to return the accelerometer. Daily counts/minute from the GT3X were uploaded to a computer to estimate total physical

activity.

• **Sedentary behaviors & recreational media use** was assessed using an instrument developed by the PACE researchers. This 9-item survey was based on a survey developed by Robinson and measures hours spent doing various sedentary behaviors during a typical weekday and weekend day (e.g., watching TV, playing computer games, doing paper work or computer work).

• **Eating Behaviors** was examined using the Weight Management Strategies Inventory, a self-report assessment of behaviors related to weight loss and weight management (e.g., self-monitoring, refusing offers of food, eating in response to emotions, exercise behaviors). The measure was developed as part of the EARLY TRIALS studies (NHLBI) by a panel of over 12 experts.

• **Well-Being** was measured by the 12-Item Short Form Survey (SF-12) that assesses 8 different health domains in physical and mental health.

• **Sleep** was assessed using 6 items that were adapted from the TREC IDEA Student Survey, Year 1, PSQI, or NHANES or created for the EARLY Trials, a NIH group of research studies.

Psychological

• **Self-esteem** was assessed with the Rosenberg Self-Esteem Scale, a well-established, 10-item questionnaire. Respondents rate their agreement on a 4-point scale with items concerning satisfaction and attitudes about their self-worth. Internal consistency coefficients have been reported to range from 0.72 to 0.84, and numerous studies demonstrate concurrent, convergent, and discriminate validity of the instrument.

• **Body image** was assessed with the body dissatisfaction subscale of the Eating Disorder Inventory – (EDI). This 9-item scale reflects the belief that specific parts of the body associated with increased 'fatness' are too large (e.g., hips, thighs, buttocks). Internal consistency and convergent and discriminant validity have been established for the scale.

• **Depression** was measured with the CES-D short form, a widely used, simple and easy to understand self-report scale. The CES-D contains 10 statements and respondents are asked to rate each statement in relation to the previous week as: "rarely or none of the time", "some or a little of the time", "occasionally or a moderate amount of time", or "most or all of the time." The CES-D has high internal consistency (= 0.8 0.9). Test-rest reliability for the short form was similar to the longer version (r = .71) and validity coefficients were in the expected direction with measures of poorer health status and negative affect.

Psychosocial Constructs

We selected a set of psychosocial measures hypothesized to mediate the intervention effect on behavior change that are consistent with the theoretical basis of the intervention and include physical activity change strategies, physical activity self-efficacy, and social support.. These measures have been developed and used in our previous studies with adults. These are generally brief measures (3 to 6 items) and have demonstrated good psychometric properties. (Robinson et al., in press & Appendices; Hagler et al., 2007).

• **Eating Self Efficacy Scale (ESES)** was used to measure controlling overeating during social and emotional situations. The ESES has 2 subscales: negative emotions and socially acceptable situations. Increases in ESES were significantly related to weight loss among weight loss participants in an original validation study.

Environmental

• **Home physical activity equipment** was assessed with a 16 item inventory developed by Sallis et al. Examples include stationary aerobic equipment, weight lifting equipment, and sports equipment, and participants are asked to indicate which items they have in their home, yard, or apartment complex. Test-retest of the summed index was .89 and scores correlated significantly with measures of PA.

• **Abbreviated Neighborhood Environment Walkability Scale** (A-NEWS) is a 53-item surveys that was developed by assessing participants' perception of their neighborhood related to being physically active.

Patterns of Physical Activity was assessed using a GPS device. A sub-sample of participants received a GPS device to wear for seven days at their baseline and 12-month measurement appointments. Collecting both location and accelerometer data allowed us to examine patterns of physical activity using our developed Physical Activity and Location Measurement System (PALMS) among participants in a weight loss study. PALMS is comprised of an integrated suite of hardware, software and database solutions that supports capture and subsequent analyses of data on PA from a geospatial perspective. Data analysis from PALMS occurred after study participants have completed the ConTxt research study.

GIS was used to create environmental measures surrounding the GPS movement tracks. Public and freely available geographically referenced data concerning roads, parks, recreation facilities, socio-economic data, land use, commercial facilities, food stores, restaurants, and green spaces was loaded into a GIS, and overlaid with participant's GPS tracks. Using a system of distance buffers from the GPS tracks, the amount and density of environmental characteristics thought to influence physical activity and eating habits was assessed for each participant's activity patterns. Additionally, time data from the GPS tracks was used to assess time spent in environments. Measures created from the GIS analysis was used to model and predict physical activity and eating habits of participants.

Acculturation was assessed using the Short Acculturation Scale for Hispanics (SASH) which is a 12-item scale that relates to 3 factors: language use, media, and ethnic social relations to assess Hispanics who are low or high in acculturation.

Implementation and satisfaction

• **Exposure to** and **satisfaction with** intervention components was assessed regularly via completion of satisfaction surveys with participants. Questions were adapted from surveys we have used in our previous work to assess web-based interventions. We also monitored the compliance of ConTxt participants to the intervention schedule, and "dose" of exposure was determined from time and date stamped electronic messages. We assessed satisfaction with the SMS/MMS messages, counseling calls, written materials.

Statistical Considerations

Power Analysis We determined sample size for a three-group design (SMS only, SMS + phone, and standard print) with percent weight loss from baseline as the primary outcome. The statistical model for the primary outcome analysis will be a random intercepts regression model. To ensure adequate power we calculated the sample size needed per treatment group for independent sample t-tests of the between group difference in percent weight loss. Because two outcome tests are of interest (SMS only vs. standard print; and SMS + phone vs. standard print) the family-wise alpha level for a two-tailed test was set at .025. Power (1 – beta) was set at .80. The effect size was estimated as d, defined as [$\mathbf{d} = | m1 - m2| / \sigma$] where m_1 and m_2 are mean change scores

of the two treatment conditions and σ is the common within-group standard deviation of the change score.

The effect size **d** was determined using results from the completed mDIET pilot study. The mDIET pilot study resulted in an estimated 2.15% between group difference weight loss (3.16% mDIET, 1.01% control) at 4 months, corresponding to a standardized effect size d of 0.52. This effect size is similar to that found in an RCT comparing a structured commercial weight loss program with a minimal self-help program. At 12-months the between group difference was 3.2% weight loss and was equal to an effect size d of .49. To detect this effect size with 80% power for the two comparisons of interest requires a sample size of 77 per group. We increased this sample size to 103 per group to allow for an attrition rate of 25% (19% loss to follow-up occurred at 4-months in the mDIET pilot study). The total sample size needed for the three-group design is N = 309.

This sample size will also give us sufficient power to test the between-group differences in the proportion of participants losing at least 5% of initial body weight. In the mDIET pilot study we found at least 5% weight loss for 36% of mDIET users compared to 12% of controls (among the 51 study completers) ((2(1) = 4.2, p = .04). With 103 participants per group we will have 80% power (with alpha = .025) to detect differences between the active interventions and the control condition in the same range (e.g., 26% vs. 10%, 33% vs. 15%, and 40% vs. 20%).

Statistical Analyses: Preliminary analyses will begin with an examination of the distribution of key variables to assess their characteristics, to provide descriptive statistics of the study population, and to allow assessment of randomization. Outliers will be assessed and variables whose distributions depart significantly from normality will be transformed. Internal consistency of multiple item scales will be examined to determine scale reliability.

Primary Outcome Analysis

As stated in the Specific Aims, the primary aim is to evaluate differences from baseline in percent weight loss at 12 months. We hypothesize that, compared to the usual care Standard Print condition, differences in weight loss will be significant in both the SMS only and SMS+Phone intervention groups.

The statistical model will be a random intercept regression model with treatment condition coded as two dummy variables (SMS only, SMS+phone and Standard Print as the reference condition) and a linear term for time (6 and 12 months). This model will include a covariate for BMI at baseline and can be expanded to determine an adjusted intervention effect by including additional covariates such as age and ethnicity. The primary tests of the active treatment conditions will be the main effects of the dummy variables representing the active treatment conditions. The two treatment x time interactions effects will further test if there is differential weight loss between 6 and 12 months between groups. This model provides maximum likelihood (ML) parameter estimates and allows for modeling of the covariance error structure of the data across the assessment points.

The primary outcome will be tested using an intent-to-treat framework. If, the extent of missing data is small and the data appear to be consistent with a missing-at-random model (MAR), then the maximum-likelihood analysis using all randomized cases and the observed data is an appropriate method for handling the missing data. In the MAR model the missingness can be a function of the observed covariates and observed outcomes. If the missingness is related to the unobserved outcome, then the missing values are considered missing not at random (MNAR). However, the MAR assumption is plausible even in studies such as substance abuse in adolescents. Graham and colleagues found that participants were more likely to leave the study for reasons such as moving away or transferring to another school than for substance abuse. The critical element

when conducting MAR-based analyses is to include variables related to the missingness in the statistical model. An important advantage of the mDIET study is that the frequent data captured through the cell phone can be used to determine study non-compliance and reasons for study withdrawal.

As a check on the sensitivity of our conclusions to the assumption that the missing data are MAR we will conduct pattern pattern-mixture modeling, which is appropriate with data is likely MNAR. In these models participants are categorized into groups based on patterns of missing data over time. Missing data pattern groups are then included as covariates in the statistical model and the effect of missing data on parameter estimates (e.g., treat x time interactions) can be determined. Parameter estimates can also be averaged over the missing data patterns to adjust for bias in the estimates.

Secondary Aims Analyses

As stated in the secondary aims, we will assess the effect of the interventions on the proportion of participants achieving at least 5% weight loss from initial body weight. A Chi-square test of proportions will be used to test the between-group difference in achieving the 5% weight loss threshold. We will conduct this test under the intent-to-treat assumption of no weight loss for cases with missing end point data and conduct a per-protocol analysis for study completers. Predictors of achieving 5% weight loss at 6 and 12 months will also be modeled using a generalized linear models with logit link function.

We will assess at 6, and 12 months the impact of the intervention on diet, physical activity, sedentary behaviors, quality of life and depression. We will first examine the relationships among these outcomes to determine if composite scores should be created from highly correlated variables. This will help to reduce the number of statistical tests.

The statistical model for each outcome will be a random intercepts regression model with treatment condition (coded as two dummy variables for SMS only, SMS+phone and Standard Print as the reference condition) and a linear term for time (0, 6 and 12 months). This model can be expanded to determine an adjusted intervention effect by including additional covariates. The primary tests of the active treatment conditions will be the two treatment x time interactions effects. In the case of discrete or skewed secondary outcome variables, generalized linear models with the appropriate link function (e.g., logit, gamma) can be estimated. These analyses will be in an intent-to-treat framework using maximum likelihood as before.

We will assess the level of satisfaction and use of Contxt components (SMS only, SMS+phone, Standard Print). Descriptive statistics (e.g., means, counts, proportions) for satisfaction and use variables will be generated. Multivariate linear regression models of these process variables will be specified to determine predictors of satisfaction and use.

We will assess differences in percent weight loss between the SMS only and the SMS+phone intervention groups. The statistical model for this analysis will be the same as the primary outcome model but will include a single indicator variable for treatment group (SMS only, SMS+phone).

Exploratory Aims Analyses

As stated in the exploratory aims, we will examine the effect of 'dose' of the intervention on outcomes at 6 and 12 months for the two active treatment conditions (SMS only, SMS+phone). We will determine appropriate markers of intervention dose (e.g., sum of SMS messages received and number of prompted responses to messages). Dose estimate variables will be included as independent variables in multiple regression models

with percent weight loss and behaviors as dependent measures adjusting for covariates. These analyses will give us information about the independent and interactive effects of the intervention components on the targeted outcomes.

We will examine mediators and Moderators of intervention effects. Following methods outlined by MacKinnon and Kraemer we will test for factors that may mediate or moderate the effect of the intervention on weight status. Intervention mediators inform how the intervention may have worked to change the outcome, while intervention moderators tell us for whom and under what conditions the intervention was most effective. Potential intervention mediators include eating behaviors, physical activity and sedentary behaviors, and changes in the home environment related to diet and activity. The statistical model will be a path analysis with regression paths from treatment condition to change in the mediator (α) and from change in the mediator to change in the outcome (β), along with the direct path from intervention to change in the outcome (τ). The statistical significance of the mediation effect ($\alpha\beta$) will be tested by dividing it by its standard error with critical values for the distribution of the product of the coefficients determined by PRODCLIN. Intervention moderators will be tested by adding interaction terms to the primary and secondary analysis models. Potential intervention moderators include gender, age category, education level, neighborhood characteristics, baseline levels of self-esteem, body image, and depression. Statistically significant interaction effects will be plotted to interpret the direction of the moderating effect of the intervention.

We will examine treatment effects among those who prefer the Spanish language ConTxt. This will be a subset analysis of those participants who indicated that they prefer speaking Spanish. The statistical model will be the same as the primary outcome analysis using a random intercept model with percent weight loss as the dependent variable.

A 25% sub-sample of participants (n=78) received a GPS device to wear for seven days at their baseline and 12-month measurement appointments. Collecting both location and accelerometer data will allow us to examine patterns of physical activity using our developed Physical Activity and Location Measurement System (PALMS) among participants in a weight loss study. PALMS is comprised of an integrated suite of hardware, software and database solutions that supports capture and subsequent analyses of data on PA from a geospatial perspective. Data analysis from PALMS will occur after study participants have completed the ConTxt research study.

10. HUMAN SUBJECTS

This is a continuation of protocol # 091040. The purpose of this protocol is for data analysis and manuscript preparation related to the study activities from protocol 091040. There is no contact (recruitment, consent, enrollment, or data collection) occurring under this new protocol. The human subject details from protocol # 091040 that are being analyzed are described below:

Focus Groups: A total of 20 p.
8-week pilot testing: A total of 20 participants (12 females, 8 males; 15 Latinos) were recruited to participate in an 8-week pilot study.
Inclusion criteria for the pilot study:

1. 21-60 years,
2. BMI of 27 – 39.9,
3. had a mobile phone and were either a current user of SMS messages or were willing and able to learn;
4. had the ability to communicate in English or Spanish.

<u>Exclusion criteria for the pilot study</u>

1. any of the following conditions: pulmonary, cardiovascular or musculoskeletal problem that would limit ability to comply with moderate intensity PA recommendations (e.g., walking),
2. were pregnant or intended to become pregnant during the study period,
3. had a history of substance abuse or other psychiatric disorder that would impair compliance with the study protocol,
4. were using medications which alter weight,
5. were enrolled in any other weight loss program.

Randomized controlled trial: A total of 298 participants aged 21-60 were recruited for the RCT. Participants were included if they have a BMI of 27-39.9, had a mobile phone and either be a current user of SMS messages or be willing and able to learn; permanent residence with the intent to stay in San Diego over the entire study period; ability to communicate in English or Spanish; and willingness to attend measurement visits at the research office. Participants were excluded from the study if they had any of the following conditions: pulmonary, cardiovascular or musculoskeletal problem that would limit ability to comply with moderate intensity PA recommendations (e.g., walking), were pregnant or intended to become pregnant during the study period, had a history of an eating disorder, substance abuse or other psychiatric disorder that would impair compliance with the study protocol, were using medications which alter weight, have had weight loss surgery (such as gastric bypass), were living with someone already enrolled in the ConTxt study, had recently quit smoking, or were enrolled in any other weight loss program.

11. RECRUITMENT AND PROCEDURES PREPARATORY TO RESEARCH

This is a continuation of protocol # 091040. The purpose of this protocol is for data analysis and manuscript preparation related to the study activities from protocol 091040. There is no contact (recruitment, consent, enrollment, or data collection) occurring under this new protocol. The recruitment details from protocol # 091040 that are being analyzed are described below:

Participants were recruited through a direct marketing approach via local media outlets including radio advertisements, print newspaper advertisements, television news stories, online newsletters, websites, flyers, and local organizations/community centers. Recruitment efforts were in both English and Spanish. Interested individuals were directed to contact the research office by phone for additional information about the study and to be screened by phone to determine if they meet the primary inclusion criteria of the study population in either English or Spanish. Verbal informed consent was obtained from potential participants.

12. INFORMED CONSENT

This is a continuation of protocol # 091040. The purpose of this protocol is for data analysis and manuscript preparation related to the study activities from protocol 091040. There is no contact (recruitment, consent, enrollment, or data collection) occurring under this new protocol. The informed consent details from protocol # 901040 that are being analyzed are described below:

Potential participants that contacted the research office had the study purpose, procedures, risks and benefits described to them. Verbal consent to participate was obtained at the time of the telephone screening. Since we anticipated receiving many calls and the screening criterion are simple and few, it was more convenient and cost-efficient for participants to complete the screening by phone. We believed that a requirement for individuals to be screened in person (with no incentive offered) would have served as a deterrent to participation and limit the representation of our sample. Written consent was obtained in person prior to beginning the study (prior to the RCT and 8-week pilot testing) and participants were given copies of the Experimental Subjects Bill of Rights in either English or Spanish.

We requested and obtained IRB approval for a waiver of written consent for the telephone screening of potential subjects for the focus groups because the telephone screening presents no more than minimal risk of harm to subjects and involves no procedures for which written consent is normally required outside of the research context.

13. ALTERNATIVES TO STUDY PARTICIPATION

This is a continuation of protocol # 091040. The purpose of this protocol is for data analysis and manuscript preparation related to the study activities from protocol # 091040. There are no human subjects involved in this protocol.

14. POTENTIAL RISKS

This is a continuation of protocol # 091040. The purpose of this protocol is for data analysis and manuscript preparation related to the study activities from protocol # 091040. All data is stored either on paper forms in locked file cabinets or on a password protected database and server. Only authorized users have access to the data. Potential risks to this study are minimal. There is a concern for confidentiality if computer or database is accessed by an unauthorized person.

15. RISK MANAGEMENT PROCEDURES AND ADEQUACY OF RESOURCES

This is a continuation of protocol # 091040. The purpose of this protocol is for data analysis and manuscript preparation related to the study activities from protocol # 091040. All data is stored either on paper forms in locked file cabinets or on a password protected database and server. Only authorized users have access to the data. Every effort will be made to maintain strict subject confidentiality when accessing the data. All information disseminated at conferences and in publications will be in aggregate form only.

16. PRIVACY AND CONFIDENTIALITY CONSIDERATIONS INCLUDING DATA ACCESS AND MANAGEMENT

All data is stored either on paper forms in locked file cabinets or on a password protected database and server. The master datasets contain study subject ID number and are stored electronically on a password protected server. The database that contains the master code is password protected and stored in a different location on a password protected servers. Only authorized users (PI, Study Coordinator, and co-investigators) will have access to that database. Every effort will be made to maintain strict subject confidentiality when accessing the data.

17. POTENTIAL BENEFITS

The overall goal of this protocol is to evaluate the effectiveness of the ConTxt Intervention on weight loss outcomes among overweight and obese adults.

18. RISK/BENEFIT RATIO

These risks are both unlikely and minor. They are reasonable in relation to the importance of knowledge that reasonably may be expected to result from the study in that the intervention may lead to weight loss among overweight and obese adults and lessons the risk of related health problems associated with overweight and obesity.

19. EXPENSE TO PARTICIPANT

This is a continuation of protocol # 091040. The purpose of this protocol is for data analysis and manuscript preparation related to the study activities from protocol # 091040.

20. COMPENSATION FOR PARTICIPATION

This is a continuation of protocol # 091040. The purpose of this protocol is for data analysis and

manuscript preparation related to the study activities from protocol # 091040.

21. PRIVILEGES/CERTIFICATIONS/LICENSES AND RESEARCH TEAM RESPONSIBILITIES

Dr. Kevin Patrick, Department of Family Medicine and Public Health, is the Principal Investigator of the project and oversee all data analysis and manuscript preparation activities.

Drs. Godino, Golaszewski, Jankowska, Yang are Co-Investigators and are contributing to analysis of the data and preparing manuscripts.

Ms. Assi is the IRB contact and will maintain IRB correspondence for this project.

Mr. Wing will be assisting with device analysis (accelerometer and GPS).

Ms. Dillon was the study coordinator.

Ms. Cameron is a MD student who is assisting with data analysis and manuscript preparations.MPH

22. BIBLIOGRAPHY

Fjeldsoe BS, Marshall AL, Miller YD. Behavior change interventions delivered by mobile telephone short message service. Am J Prev Med 2009; 36(2): 165-73.

Hurling R, Catt M, Boni MD, Fairley BW, Hurst T, Murray P, et al. Using internet and mobile phone technology to
deliver an automated physical activity program: randomized controlled trial. J Med Internet Res 2007; 9(2): e7.

Joo NS, Kim BT. Mobile phone short message service messaging for behaviour modification in a community-based weight control programme in Korea. J Telemed Telecare 2007; 13(8): 416-20.

NIDDKD. Overweight, obesity, and health risk. National Task Force on the Prevention and Treatment of Obesity. Arch Intern Med 2000 Apr 10; 160(7): 898-904.

Tate DF, Wing RR, Winett RA. Using Internet technology to deliver a behavioral weight loss program. JAMA 2001 Mar 7; 285(9): 1172-7.

23. FUNDING SUPPORT FOR THIS STUDY

This is a continuation of protocol # 091040. The purpose of this protocol is for data analysis and manuscript preparation related to the study activities from protocol # 091040. Protocol # 091040 was funded by a grant from the National Institutes of Health. This new protocol is unfunded.

24. BIOLOGICAL MATERIALS TRANSFER AGREEMENT

N/A

25. INVESTIGATIONAL DRUG FACT SHEET AND IND/IDE HOLDER

N/A

26. IMPACT ON STAFF

N/A

27. CONFLICT OF INTEREST
N/A
**28. SUPPLEMENTAL INSTRUCTIONS FOR CANCER-RELATED STUDIES**
N/A
**29. OTHER APPROVALS/REGULATED MATERIALS**
N/A
**30. PROCEDURES FOR SURROGATE CONSENT AND/OR DECISIONAL CAPACITY ASSESSMENT**
N/A
